# Genome-Wide Characterization Reveals Variation Potentially Involved in Pathogenicity and Mycotoxins Biosynthesis of *Fusarium proliferatum* Causing Spikelet Rot Disease in Rice

**DOI:** 10.3390/toxins14080568

**Published:** 2022-08-19

**Authors:** Ling Wang, Shuailing Ge, Wenhao Liang, Weiyang Liao, Wen Li, Gui’ai Jiao, Xiangjin Wei, Gaoneng Shao, Lihong Xie, Zhonghua Sheng, Shikai Hu, Shaoqing Tang, Peisong Hu

**Affiliations:** State Key Laboratory of Rice Biology, China National Center for Rice Improvement, China National Rice Research Institute, Hangzhou 311401, China

**Keywords:** *Fusarium proliferatum*, spikelet rot disease, rice, genome, pathogenicity, mycotoxins

## Abstract

*Fusarium proliferatum* is the primary cause of spikelet rot disease in rice (*Oryza sativa* L.) in China. The pathogen not only infects a wide range of cereals, causing severe yield losses but also contaminates grains by producing various mycotoxins that are hazardous to humans and animals. Here, we firstly reported the whole-genome sequence of *F. proliferatum* strain Fp9 isolated from the rice spikelet. The genome was approximately 43.9 Mb with an average GC content of 48.28%, and it was assembled into 12 scaffolds with an N_50_ length of 4,402,342 bp. There is a close phylogenetic relationship between *F. proliferatum* and *Fusarium *fujikuroi,** the causal agent of the bakanae disease of rice. The expansion of genes encoding cell wall-degrading enzymes and major facilitator superfamily (MFS) transporters was observed in *F. proliferatum* relative to other fungi with different nutritional lifestyles. Species-specific genes responsible for mycotoxins biosynthesis were identified among *F. proliferatum* and other *Fusarium* species. The expanded and unique genes were supposed to promote *F. proliferatum* adaptation and the rapid response to the host’s infection. The high-quality genome of *F. proliferatum* strain Fp9 provides a valuable resource for deciphering the mechanisms of pathogenicity and secondary metabolism, and therefore shed light on development of the disease management strategies and detoxification of mycotoxins contamination for spikelet rot disease in rice.

## 1. Introduction

Spikelet rot disease in rice (*Oryza sativa* L.) is disease that has been emerging over recent years in China. It is widespread along the middle and lower reaches of the Yangtze River, due to the susceptibility of cultivated *japonica* varieties and *indica*-*japonica* hybrids. The initial symptoms of the disease are characterized by reddish-brown or rust-red spots on the glumes at the flowering and milking stage, then become blackish-brown at the ripening stage. Affected spikelets are partially filled or aborted and therefore do not produce edible grains. The average yearly occurrence of spikelet rot disease in rice affects more than 800,000 hectares in China. In warm and rainy regions, the prevalence of spikelet rot disease dominates, and yield losses can reach up to 30% in the field. In order to decrease damage of spikelet rot disease in rice, the application of fungicides is a primary control measure when weather conditions are conducive to fungal infection. However, the effectiveness of fungicides varies and is strongly dependent on different factors, such as the resistance of the rice cultivar, disease severity, and spraying technology.

The filamentous ascomycete *Fusarium proliferatum* (Matsushima) Nirenberg [teleomorph: *Gibberella fujikuroi* (Kuhlman) Samuels], belonging to *Fusarium* section *Liseola*, was reported for the first time in 2011 as the primary cause of spikelet rot disease in rice in China [1]. The fungus is a notorious phytopathogen, not only resulting in devastating diseases on diverse agricultural crops, such as wheat, rice, barley, and maize [2], but also producing a wide spectrum of toxic secondary metabolites (SMs) or mycotoxins, including fumonisins, moniliformin, fusaric acid, fusarin C, and beauvericin, which can accumulate in food and feed crops and therefore pose risks to human and animal health. Especially the production of fumonisin mycotoxins has received greater attention than other mycotoxins. High levels of fumonisins are epidemiologically associated with adverse health effects, including leukoencephalomalacia in horses, pulmonary edema in swine, hepatotoxicity in rats, nephrotoxicity in rodents, and cancer of the esophagus as well as neural tube defects in humans [3]. Some evidence founded that fumonisins production by *F. proliferatum* as a potential virulence factor was required for development of disease symptoms on hosts [4,5]. To minimize human and animal exposure to mycotoxins produced by *F. proliferatum*, including fumonisins, moniliformin, beauvericin, and fusarins, regulatory organizations have established maximum permissible levels in cereals and the products by the European Union, the joint Food and Agriculture Organization (FAO), and World Health Organization (WHO) [6,7].

Despite the potential importance concerning economic impact or human health, the lifestyle of *F. proliferatum* remains poorly documented and is still required to be extensively investigated. Genome decoding is the first step to decipher the range of molecular basis driving genetic variability, host–pathogen interactions, and the regulation of secondary metabolism. However, our knowledge of the complete gene set of *F. proliferatum* still remains incomplete. To date, only a few whole-genome sequences are publicly available of *F. proliferatum* strains, the strain ITEM 2341 from date palm [8], the strain A8 from onion [9], strain ET1 from orchid, and strain NRRL 62905 from maize [10]. Further expansion of the genomic resources of *F. proliferatum* provides a basis for our understanding of the pathogenesis and toxin biosynthesis of this pathogen.

As rice is an important crop used for human consumption, its safety is of concern. Moreover, increasing evidence suggested that fungicides might not be efficient at reducing mycotoxins contamination [11]. Hence, comprehensive genetic analyses are needed to provide knowledge focusing on morphology, pathology, and toxigenicity from the fungal aspect to develop the practical intervention strategies of mycotoxins decontamination. In this study, we first report a high-quality draft genome for *F. proliferatum* isolated from rice. We then approximated the repertoire of the genes and characterized structural features of the genome. Then, we evaluated the genetic components involved in pathogenicity and secondary metabolism, with a particular emphasis on the main gene families and the biosynthetic gene clusters of mycotoxins. The results not only gave insight into the genetic architecture of *F. proliferatum* but also contributed to strategies for reduction of crop diseases and mycotoxin contamination, which could also impact positively on food safety and security.

## 2. Results

### 2.1. Genome Sequence Assembly

The monoconidial strain Fp9 of *F. proliferatum* was isolated from an infected spikelet of rice (cv. Xiushui 09). The strain Fp9 was confirmed as *F. proliferatum* based on the concatenated alignment of the nucleotide sequences of *ITS*, *TEF-1α*, and *β-TUB* genes with MEGA (Figure S1).

**Figure 1:**
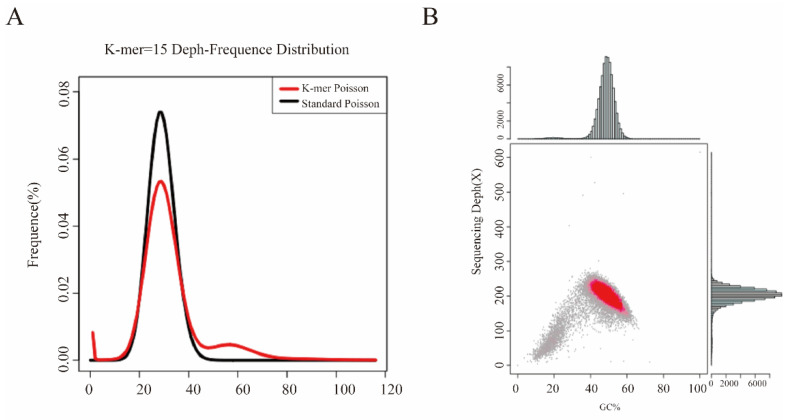
The analyses of k-mer distribution and GC depth distribution for Illumina sequencing data in *F. proliferatum*. (**A**) Fifteen k-mer depth distribution of whole-genome Illumina reads. (**B**) The relationship between GC content and sequencing depth of the genome data used for assembly. The average sequencing depth was detected at near 120-fold coverage of the genome.

The genome of the strain Fp9 was sequenced using massively-parallel-sequencing Illumina technology. One paired-end library with an average insert size of 400 bp was established to obtain clean reads of 5953 Mb, and two mate-pair libraries with insert sizes of 2 kb and 6 kb were constructed to obtain clean reads of 3906 Mb and 4156 Mb, respectively. The error-corrected genome sequences were generated by mapping Illumina short reads to long reads. After discarding low-quality reads and adaptor reads, a total of 43,919,396 reads were obtained corresponding to approximately 120× sequence depth (Figure 1). The filtered sequences were assembled by SOAPdenovo into 190 contigs with N_50_ length of 730,687 bp and N_90_ length of 210,543 bp, respectively. The contigs were further assembled into 12 scaffolds with N_50_ length of 4,402,342 bp and N_90_ length of 2,484,728 bp, respectively. Accordingly, the estimated genome size was 43.9 Mb with an average GC content of 48.28%. The draft genome assembly had 98.4% and 99.2% completeness with the ascomycota_odb10 dataset (*n* = 1706) and fungi_odb10 dataset (*n* = 758), respectively, according to the evaluations with BUSCO, which suggests a high degree of completeness for the genome assembly. The genome sequence had been deposited in the GenBank database under the accession number WKFO00000000 (PRJNA517537 for BioProject and SAMN10829574 for BioSample).

### 2.2. Genome Comparison and Phylogenetic Relationship

**Figure 2:**
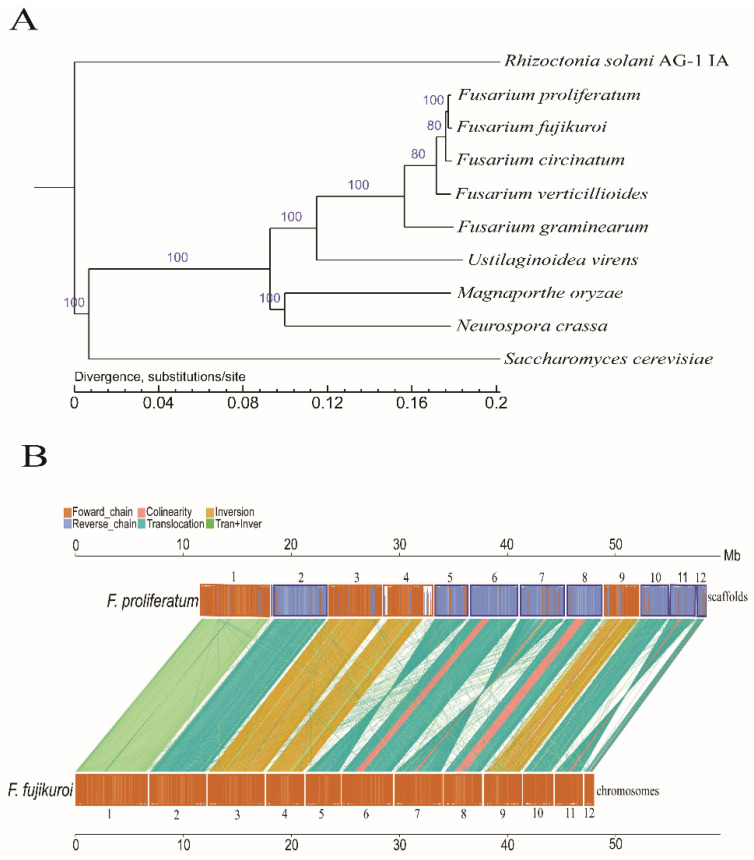
The phylogenomic relationship of *F. proliferatum* with other filamentous fungi. (**A**) Phylogenetic tree of *F. proliferatum* and other nine selected fungal species (eight Ascomycota and one Basidiomycota). Basidiomycota fungus *Rhizoctonia solani* AG-I IA was used as the outgroup. Bootstrap values were calculated from 1000 replicates and marked in each note. (**B**) A whole-genome alignment between *F. proliferatum* and *F. fujikuroi*. The synteny of nucleotide sequences was indicated by vertical lines. In the upper and lower axes, the brown frame represented the genome forward chain, the blue frame represented the genome reverse chain, and the height of the color filled in the frame represented the similarity degree of the comparison. The color comparison types of the linked graph between the upper and lower axes were as follows: Collinear (red), Translocation (green), Inversion (yellow), and Tran + Inver (light green).

Phylogenetic analysis for the evolutionary relationship between the *F. proliferatum* strain Fp9 and other fungal species (eight ascomycota and one basidiomycota outgroup) showed that *F. proliferatum* was most closely related to *Fusarium fujikuroi,* the causal agent of the bakanae disease of rice (Figure 2A). Despite the shared genomic synteny in both genera (Figure 2B), it is noteworthy that the large fragments containing species-specific genes were significantly enriched in the subtelomeric regions, e.g., 56.2-kb and 24.6-kb, and inserts were present in far end of the left and right arms of scaffold IV in *F. proliferatum* with respect to *F. fujikuroi* (Table S1). Functional enrichment analysis using the MIPS ‘FunCat’ program revealed that subcategories of the ‘oxidation-reduction process’ was remarkably enriched in *F. proliferatum* and underrepresented in *F. fujikuroi*, whilst genes annotated as being integral to membrane, nucleic acid binding, zinc ion binding, regulation of transcription, and metabolic process were significantly higher in *F. fujikuroi* than in *F. proliferatum* (Table S2). To validate structural variants (SVs) between *Fusarium proliferatum* and *Fusarium fujikuroi*, 20 SVs were randomly selected for performing PCR amplification and paired-end Sanger sequencing. Each amplification gave the product size expected from the event inferred in silico (Table S3, Figure S2). The sequencing of amplified products likewise confirmed the inferred events in each case (Additional File S1).

### 2.3. Expansion of Prominent Gene Families

A total of 14,054 protein-coding genes were predicted for the genome of *F. proliferatum* strain Fp9 by GeneMarkS with integrated model. For the functional annotations of predicted genes, all genes were interrogated into the known databases. Among the predicted coding genes, 13,798 (98.18%), 4269 (30.38%), 2226 (15.84%), 2085 (14.84%), 3279 (23.33%), and 13,248 (94.26%) genes had homologs with known functions in the Non-Redundant Protein Database databases (NR), Kyoto Encyclopedia of Genes and Genomes (KEGG), Clusters of Orthologous Groups (COG), Gene Ontology (GO), Swiss-Prot, and TrEMBL databases, respectively.

As a fungal plant pathogen, 1591 (11.32%) genes were considered to be involved in interaction between the pathogen and host, according to the best-matched with the pathogen–host interaction database (PHI-base). A total of 1222 (8.70%) genes harboring N-terminal signal peptides were predicted to produce the secreted proteins using SignalP 5.0. The secreted protein-encoding genes were thought to be one of the essential components of aggressiveness, which randomly distributed across the genome of *F. proliferatum*, with no evidence of clustering on particular chromosomes as had been reported for *Fusarium oxysporum* [12]. We found that 1402 (9.98%) genes were predicted to be membrane transporters in *F. proliferatum* as indicated by Transporter Classification Database, which surpassed the number of most other ascomycetes (Table S4). *F. proliferatum* encoded considerably major facilitator superfamily (MFS), which was the largest classes of membrane transporters.

**Figure 3:**
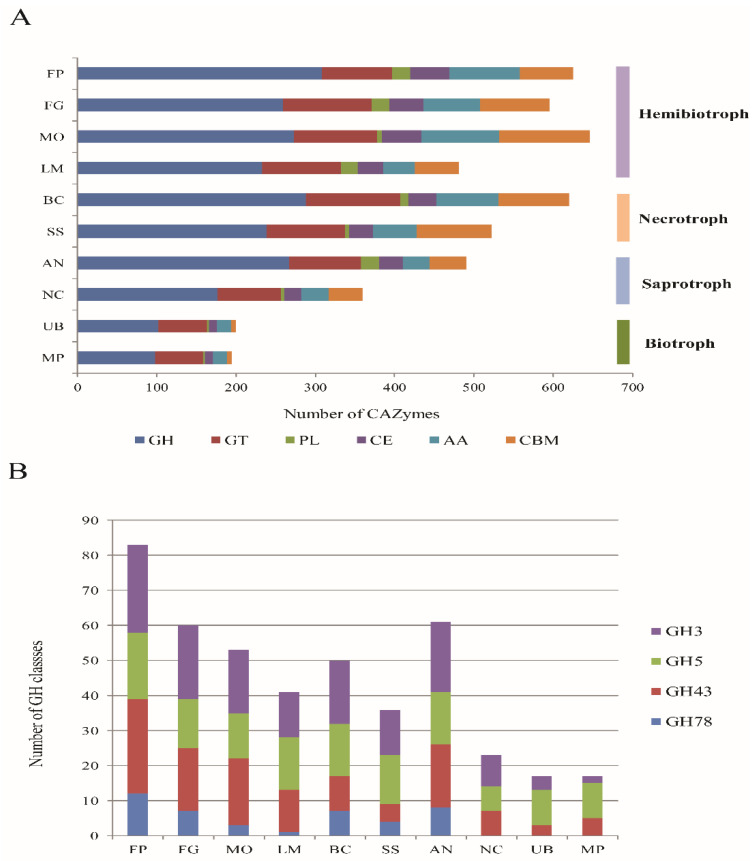
Carbohydrate-active enzymes (CAZymes) significantly enriched in *F. proliferatum* relative to other fungi with various lifestyles. (**A**) Number of fungal CAZyme families of *F. proliferatum* and other fungal species. Hemibiotroph: FP, *Fusarium proliferatum*; FG, *Fusarium graminearum*; MO, *Magnaporthe oryzae*; LM, *Leptosphaeria maculans*. Necrotroph: BC, *Botrytis cinerea*; SS, *Sclerotinia sclerotiorum*; Saprotroph: AN, *Aspergillus nidulans*; NC, *Neurospora crassa*. Biotroph: UB, *Ustilago bromivora*, MP, *Melanopsichium pennsylvanicum*. Families: GH, glycoside hydrolases; GT, glycosyl transferase; PL, polysaccharide lyase; CE, carbohydrate esterase; AA, auxiliary activities enzymes; CBM, carbohydrate binding module. (**B**) Comparison of GH repertoires of *F. proliferatum* and other fungal species. Families GH3 and GH5 encode cellulose-degrading enzymes, family GH43 encodes both cellulose and pectin-degrading enzymes, and family GH78 encodes pectin-degrading enzymes.

Additionally, 625 (4.45%) genes encoding putative carbohydrate-active enzymes (CAZymes) were identified with the CAZymes database, of which, 308 glycosyl hydrolases (GHs), 89 glycoside transferases (GTs), 23 polysaccharide lyases (PLs), 49 carbohydrate esterases (CEs), 89 auxiliary activities enzymes (AAs), and 67 carbohydrate-binding modules (CBMs) (Table S4). The CAZymes repertoires of *F. proliferatum* were similar to those of the hemibiotrophic and necrotrophic fungi, i.e., *Magnaporthe oryzae*, *Fusarium graminearum*, and *F. fujikuroi*, but more expanded than those found in the saprotrophic and biotrophic fungi (Figure 3A). Particularly, some families of carbohydrate and non-carbohydrate substrates (GHs) and pectin-based components (PLs), such as GH3, GH5, GH43, and GH78, were enriched in *F. proliferatum* compared with other fungi representing various nutritional lifestyles (Figure 3B, Table S5).

### 2.4. Diversification of the Biosynthetic Gene Clusters for Mycotoxins

To estimate the genetic potential of *F. proliferatum* to produce SMs, genes encoding key classes of SM-associated enzymes were predicted. Altogether, there were 778 genes present in 57 gene clusters in *F. proliferatum*, which catalyzed synthesis of molecules that serve as core structures for secondary metabolites, i.e., fifteen polyketide synthases (PKSs), eleven nonribosomal peptide synthetases (NRPSs), five hybrid PKS-NRPS, fourteen terpenes, one lantipeptide, as well as eleven other clusters. A genome-wide comparison of gene clusters among *Fusarium* genomes showed that most of the gene clusters of *F. proliferatum* were typically conserved compared to other *Fusarium* species, whilst some of SM genes had marked differences in gene content of homologous clusters.

**Figure 4:**
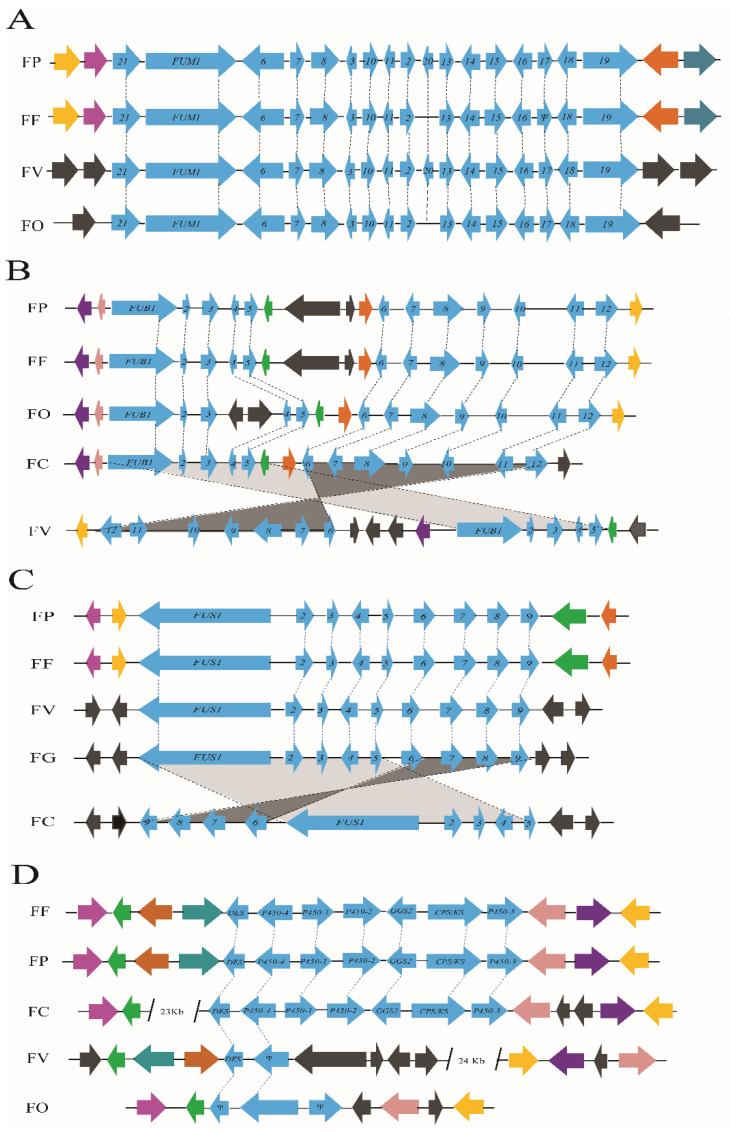
Comparison of the biosynthetic gene clusters of secondary metabolites in *F. proliferatum* with homologous regions in other *Fusarium* species. (**A**) Content and arrangement of the fumonisin biosynthetic gene (*FUM*) cluster in *Fusarium* species. (**B**) Content and arrangement of the fusaric acid gene (*FUB*) cluster in *Fusarium* species. (**C**) Content and arrangement of the fusarin C gene (*FUS*) cluster in *Fusarium* species. (**D**) Content and arrangement of the gibberellin gene (*GA*) cluster in *Fusarium* species. Organisms: FP, *Fusarium proliferatum*; FF, *Fusarium fujikuroi*; FV, *Fusarium verticillioides*; FO, *Fusarium oxysporum*; FG, *Fusarium graminearum*; FC, *Fusarium circinatum*. The genes considered to be part of the gene clusters were represented by horizontal blue arrows. The same colored arrows represented homologous genes in two or more fungi. Black arrows indicated that the gene did not have a closely-related homolog in the SM cluster of the species examined. The Greek letter Ψ indicated a pseudogene. The arrowheads indicated the direction of transcription of the gene.

The content and arrangement of the fumonisin gene (*FUM*) cluster in *F. proliferatum* was very similar to that in *F. fujikuroi* and *Fusarium verticillioides*, and the *FUM* cluster was arranged in a highly collinear manner in the three fusaria (Figure 4A). Although the gene order and orientation of *FUM* cluster in *F. proliferatum* were the same as those in *F. verticillioides*, there was striking difference in the cluster flanking genes between them. The fusaric acid gene (*FUB*) cluster showed collinearity among fusaric acid (FA)-producing fusaria, but the homologous segments were separated by three or four genes which were not involved in FA biosynthesis, and the *FUB* genes exhibited a discontinuous distribution (Figure 4B). The genomic locations of orthologous genes of *FUB* cluster in *F. verticillioides* were inverted in comparison to *F. proliferatum*. In contrast to the *FUB* cluster, the biosynthetic gene clusters of the bikaverin and fusarubin, which are responsible for production of the PKS-derived perithecial pigmented naphthoquinone, were almost perfectly maintained with high levels of similarity of gene content, and flanking genes at least one side were largely collinear amongst the members of *Gibberella fujikuroi* species complex (GFC) (data not shown). The biosynthetic gene cluster of apicidin F was present in the genome sequence of *F. fujikuroi* but absent from that of the highly-related species *F. proliferatum* and the other *Fusarium* species examined, e.g., *F. verticillioides*, *F. oxysporum*, or *Fusarium circinatum*.

The flanking regions of the fusarin C gene (*FUS*) cluster do not exhibit homology among *Fusarium* species, and *FUS* genes were reversed in *F. circinatum* compared to *F. proliferatum* (Figure 4C). The flanking genes of the *FUS* cluster were arranged markedly different among *Fusarium* genomes examined. The homologs of the entire gene cluster for gibberellin (GA) biosynthesis in *F. fujikuroi* were also found in *F. proliferatum* (Figure 4D). *F. circinatum* had one homolog of the GA cluster, whereas the gene content of the flanking regions was absent. In contrast, *F. verticillioides* and *F. oxysporum* lacked GA clusters but had partial, nonfunctional homologs that were present in a similar syntenic region as the GA cluster in *F. fujikuroi*.

## 3. Discussion

In recent years, spikelet rot disease in rice has been considered an emerging disease even where it was previously absent or scarce in China. Management efforts using resistant varieties and/or fungicides were inadequate for disease control due to mycotoxins contamination. Given its economic and public health importance, the causal agent *F. proliferatum* has been attracted greater attention. The availability of genome sequences has facilitated in the understanding of genome composition and host virulence as well as the biosynthetic capacity of natural compounds. Although there were several published genome databases of *F. proliferatum*, these were not sampled from rice fields. Here, we presented a genome of *F. proliferatum* stain Fp9 collected from rice spikelet in the fields, which allowed us to make the comprehensive investigation of the genetic bases of *F. proliferatum*.

In the current study, next-generation sequencing (NGS) technology was applied to generate the *F. proliferatum* reference genome. The high quality genome sequence of strain Fp9 that was assembled into twelve scaffolds was used for structural annotation and comparative analysis. However, NGS technologies (such as Illumina, 454, and Ion-torrent) have shortcomings in their capability to investigate repetitive elements, structural variants, or native base modifications [12,13]. Several of the limitations can be overcome by third-generation sequencing technologies (TGS). The advent of TGS technologies, such as the Pacific Biosciences (PacBio) and Oxford Nanopore machines, provides new possibilities for contig assembly, scaffolding, and high-performance computing in bioinformatics due to its long reads [14]. The high level of error can be reduced to <1% with the PacBio technology with circular consensus long read (CCS) approach [15]. NanoVar generated by Nanopore technologies exhibits higher structural variant calling accuracy using low-depth simulated datasets [16]. For projects where both genome continuity and consensus accuracy matters, a combination of longer reads and transcriptome analysis may be needed to obtain the accurate and realistic genome of *F. proliferatum* in the future studies.

The phylogenetic analysis revealed the high synteny between *F. proliferatum* and *F. fujikuroi*, the causative agent of the bakanae disease of rice. Abundant species-specific genes were found near the subterminal regions. Variation in the proximal telomere was also characterized in *Aspergillus* species by the expansion and deletion of the unique genes with a bias towards subtelomeric locations [17]. Such phenomenon is common in fungal pathogens and more generally in eukaryotic genomes. In fungi, gene loss and changes in genomic architecture are important adaptation processes, which may be a consequence from their parasitic lifestyle that pushes their need to occupy an ecological niche [18]. Without selective pressure, the higher speed of diversification in telomeric-like and subtelomeric-like regions had been already reported in the genome of *F. graminearum* [19]. Codon usage indices suggested that subtelomeric genes use a lower frequency of optimal codons and display a lower codon adaptation index with a higher effective number of codons [20]. Our analyses provided the support for the hypothesis that species-specific genes were concentrated in subtelomeric regions that led to genetic divergence between *F. proliferatum* and *F. fujikuroi*. The most abundant category of *F. proliferatum*-specific genes with a known function that was associated with oxidoreductase, which are likely be related to several important facets of fungal biology, such as cell differentiation, growth, and reproduction [21]. As a follow up to this study, we will focus on specific genes that benefit potential contribution by conferring adaptive metabolic advantages in the evolution of *F. proliferatum*.

MFS transporters are currently the largest characterized superfamily of transmembrane secondary transport proteins, which are responsible for nutrient uptake, metabolite extrusion, and resistance to various toxic compounds, including not only secondary metabolites but also fungicides and antibiotics [22]. Most significantly, MFS transporters were enriched in *F. proliferatum*, that likely cooperate and collectively contribute to mycotoxin efflux, as the self-defense of MFS transporters against deoxynivalenol (DON) in *F. graminearum* [23]. Intriguingly, MFS transporters could function directly to avoid the toxicity of chemicals by pumping them out of the cells, thus provided defense against toxic oxidants and acted as a virulence factor by protecting the fungi from toxic compounds [24]. On the other hand, MFS transporters play a role in nutrient availability for the survival, including the transport of lipids, ions, and small metabolites, as previously implicated in *Botrytis cinerea* [25]. Therefore, the large number of MFS transporters in the genome of *F. proliferatum* might involve in nutrients uptake from hosts to survive in the first site of infection, and detoxification in counteracting the physiological impact of antimicrobial host defense compounds to thrive in stressful host niche colonization sites.

In order to breach plant barriers for invasion and colonization, the phytopathogenic fungi were equipped with abundant and diverse enzymes for the degradation of plant polysaccharide materials. In this study, a comparison of the abundance of the full repertoires of CAZymes was performed among saprotrophic, necrotrophic, hemibiotrophic, and biotrophic fungi. We found that hemibiotrophic and necrotrophic fungi tended to have more CAZymes than biotrophic and saprotrophic fungi. As a hemibiotroph, *F. proliferatum* undergoes a shift in lifestyle from biotrophy to necrotrophy during the infection process. The CAZymes families related to the hydrolytic activity of celluloses (GHs) and the oxidative degradation of pectins (PLs) were more prevalent in *F. proliferatum* than other test fungi with different nutritional modes. As mentioned previously, GHs and PLs are often known as cell wall-degrading enzymes (CWDEs) due to their important roles in plant biomass decomposition by fungal pathogens [26]. Hemibiotrophic fungi obtain nutrition for growth and development mainly by the localized degradation of cell walls with a variety of CWDEs. Such expansion of CWDEs could explain, at least in part, the capacity of *F. proliferatum* for the penetration and successful infection of the hosts due to high cellulolytic, hemicellulolytic, and pectinolytic abilities. As a matter of fact, epidemic spreading across multiple hosts were caused by introductions of pathogen from restricted geographic regions, as reported for *Phytophthora infestans* [27]. The broad host-range pathogens were obviously more prone to rapidly respond to the environmental condition of a new habitat [28]. For spikelet rot disease in rice, the disease prevalence was likely relevant to multiple host adaptability of *F. proliferatum*. Clarifying the specific biological functions of CAZymes participating in the interactions with hosts, which likely provide information for a better understanding of its pathogenic process and host colonization.

Hemibiotrophy includes the two sequential stages of infection, biotrophy, and necrotrophy, in a series of steps that involve the participation of different virulence factors [29]. In *Colletotrichum higginsianum* infecting *Arabidopsis thaliana* and *Colletotrichum graminicola* infecting maize, comparative genomics showed that both fungi had large sets of pathogenicity-related genes, but families of genes encoding secreted effectors, pectin-degrading enzymes, secondary metabolism enzymes, transporters, and peptidases were expanded [30]. *Ustilaginoidea virens* (Cooke) Takah is an ascomycetous fungus that causes rice false smut, possessed reduced gene inventories for polysaccharide degradation, nutrient uptake, and secondary metabolism, which may play essential roles in a biotrophic lifestyle and adaptation to the specific floret infection [31]. In *Penicillium digitatum*, genes encoding plasma membrane transporters that may be required for assimilating major polysaccharides in the host wall, for example, oligopeptides, amino acids, and sugars, are also induced [32]. The rich sources of MFS transporters and CWDEs in *F. proliferatum*, suggested the pathogen required a maximum capacity for host manipulation during intracellular colonization, and biotrophic hyphae could provide the uptake of sugars and amino acids, which might be an adaptation to exploit a broad range of host plants by its multistage hemibiotrophic infection strategy. Major hemibiotrophic plant pathogens, such as the rice blast fungus *Magnaporthe oryzae* and charcoal rot fungus *Macrophomina phaseolina*, undergo major transformations in cell morphology and infection mode when switching from growth on the plant surface to intracellular biotrophy and from biotrophy to necrotrophy [33,34]. In this case, *F. proliferatum* invades the host cell in the biotrophic stage, then produces cellulolytic enzymes to hijack host metabolic pathways for a better uptake of plant nutrients by transporters during the necrotrophic stage, and finally, the fungus differentiates fast-growing hyphae that kill and destroy host issues. MFS transporters and CWDEs in *F. proliferatum* may thus be particularly important for the ability of the fungus to rapidly adapt to changing environments and occupy a wide range of habitats, including a diversity of crop plants.

In filamentous fungi, SMs are usually regarded as not essential for fungal growth and development, but they fulfill various functions, such as virulence factors, chemical signals for communication, defense against competitors, and symbiotic interactions [35]. Fungal SM biosynthetic genes were typically located in clusters for common gene regulation. Several studies had explored the evolutionary drivers of divergence of SM genes, including point or indel null mutations in the core genes, gene-conversion, inversion, and duplication/deletion, gene rearrangements and HGT events [36,37,38]. The species of the fungus *Fusarium* were known for the potential to synthetize structurally distinct SMs and some were known to be involved in playing roles in triggering host cell death and disease development [39,40]. The comparison of the genome of *F. proliferatum* to those of other fusaria revealed that most of SMs biosynthetic genes were relatively conserved. However, several gene clusters displayed multiple and disparate patterns of distribution, which exhibited the divergence of metabolites in the genus *Fusarium*. A similar situation was found for the aflatoxin gene cluster in the genus *Aspergillus* section Flavi, some of the species produced aflatoxin (e.g., *A. flavus, A. parasiticus,* and *A. nomius*), whereas others had lost the ability for aflatoxin production (e.g., *A. oryzae* and *A. sojae*) [41]. It appeared that structure and distribution of SM gene clusters contributed to metabolic diversity of the fusaria. However, only few gene clusters responsible for the natural products were already well characterized, probably due to the fact that most are not or only minimally expressed under standard laboratory conditions [42]. Furthermore, comprehensive analyses are required to fully understand the regulation of secondary metabolism in the fusaria.

The genus *Fusarium* can potentially synthesize harmful mycotoxins deriving from secondary metabolism, as exemplified by fumonisins, trichothecenes, zearalenone, moniliformin, and beauvericin. The economic impact of *Fusarium*-induced mycotoxin contamination via its effects on health of humans and livestock as well as on international trade was estimated to be in the hundreds of millions of US dollars every year [43]. The examination of the genetics of mycotoxins biosynthesis is motivated by the need to limit mycotoxin contamination of food and feed and to understand how mycotoxins impact pathogen-infected crops. In the current study, the composition and arrangement of biosynthetic gene clusters involved in the known mycotoxins production of *F. proliferatum* and other fusaria were analyzed in more detail.

The analyses of cluster flanking regions provided evidence that the functional homologs responsible for fumonisin production were located at different genomic position in *F. proliferatum* relative to *F. verticillioides*, which supported the hypothesis that the *FUM* cluster was acquired by horizontal gene transfer (HGT) from a progenitor homolog [44]. In *F. verticillioides*, fungi can expand its metabolic capabilities in such a way that, in turn, enhanced the adaptive strategies [45]. It had been shown that an intact *FUM* cluster also existed in *Aspergillus niger* [46]. Synonymous site divergence between the *A. niger* and *F. verticillioides* highlighted how the *FUM* cluster can diverge through the rearrangement of gene positions or by addition or loss of genes within *FUM* cluster [47]. Nevertheless, the presence of the *FUM* gene cluster did not always correlate with the ability to produce fumonisins. In contrast to the high amounts of fumonisin production presenting in the cracked maize kernel culture of *F. proliferatum* strain ITEM 2287 and *F. verticillioides* strain NRRL 20956, the main fumonisins producers among the genus *Fusarium*, no or relatively small amounts of fumonisins was observed in *F. fujikuroi* strains GL24 and GL28 [44]. Possible reasons could be that *FUM20* was absent and *FUM17* was present as a pseudogene that caused a premature truncation in *F. fujikuroi*.

The gene cluster responsible for fusaric acid exhibited a discontinuous distribution among *Fusarium* species, displaying gene insertion/deletion, inversion, or translocations. A possible explanation for this finding was that multiple independent events of genome rearrangements occurred within *FUB* gene clusters as species diverged from each other. The presence of the non-*FUB* genes within *FUB* cluster among *Fusarium* genera could lead to analogs of metabolites that had the similar core structure but differ markedly in modifications to the structure [48], as was the case for structural diversity of trichothecenes in *Fusarium* [49]. However, trichothecene biosynthetic (*TRI*) genes were organized into three segments, the *TRI*-gene segments differed from the *FUB*-gene segments in that they were distinct loci, and in *F. graminearum*, each of these *TRI* loci was on a different chromosome [50]. In *F. verticillioides*, the *FUB1* to *FUB5* and *FUB6* to *FUB12* segments of the *FUB* cluster underwent inversion relative to the homologous segments in other *Fusarium* species, which suggested that genome rearrangements were another potential cause of the phylogenetic incongruence and the deep divergence of the cluster sequences of fusaric acid.

The most studied NRPSs in *Fusarium* species is the one responsible for the biosynthesis of Apicidin F [51]. Most members of *Fusarium* species exhibited small or large deletions in the biosynthesis genes that were responsible for apicidin F. It was most likely that an ancestor of the genus *Fusarium* maintained the entire gene cluster of apicidin F in genome [52], which had been lost or retained remnants of cluster paralogues in some species during evolutionary history. Although the biosynthetic gene cluster of apicidin F was present in the genome sequence of *F. fujikuroi*, additional genome sequences of *F. fujikuroi* showed that this cluster was only present in strain B14 but not present in other strains of this species [20].

The first fungal PKS-NRPS to be characterized was the fusarin C synthetase [53]. Although the order and orientation of the *FUS* cluster responsible for fusarin C production was largely conserved among the *Fusarium* species examined, the flanking regions were arranged markedly different, which likely reflected that the *FUS* cluster had undergone multiple independent rearrangement events associated with translocation and inversion, e.g., via HGT events. A functional full-length homolog of *FUS* cluster was required for synthesis of fusarin mycotoxins. However, the deletion events of *FUS* cluster had likely occurred. For example, *F. proliferatum* from a variety of hosts and geographic locations indicated that 33 of the strains had an intact *FUS* cluster, and only three isolates had the partially deleted *FUS* cluster [54]. A possible explanation for this finding was that the partial deletion of the *FUS* cluster occurred in a common ancestor of *F. proliferatum* in such a way that it resulted in two alleles of the *FUS* cluster.

Gibberellins (GAs) belong to a large family of tetracyclic diterpenoid carboxylic acids, and some members of them are phytohormones. GAs were first isolated as secondary metabolites of the pathogenic fungus *F. fujikuroi* in rice seedings. Complete and partial homologs of the GA clusters existed in some *Fusarium* species, although no GA or GA-like compounds had been reported in these species [42]. The lack of GA production in other fusaria could be caused by a number of factors, including mutations that leave ORFs intact but render enzymes nonfunctional, the reduced transcription of GA genes, improper GA transcript processing, and/or altered translation [42]. *F. proliferatum* lost the ability to produce GAs due to mutations in *P450*-*1* and *P450*-*4* genes that lead to premature termination of the predicted ORF [55]. In *F. avenaceum*, a GA gene cluster with homologs of six of the seven GA biosynthetic genes was found, the homologs lacked a *P450-3* homolog encoding the 13-hydroxylase [56]. In *F. fujikuroi*, the P450-3 enzyme catalyzed the last step of the conversion of GA7 to GA3 in GA biosynthetic pathway [57]. It is noteworthy that although GA biosynthetic genes were present in some related species, the ability to produce Gas and express GA biosynthetic genes was limited to *F. fujikuroi* [58].

On the whole, the biosynthesis of mycotoxins was complex among members of the *Fusarium* species. Mutations, HGT events, and genome reorganization, as well as gene loss and gene gain, could directly influence the re-shuffling of biosynthesis genes of mycotoxins, which probably led to synthesize putatively novel derivatives or lack the corresponding metabolic capability. Unique biosynthetic clusters of mycotoxins in *F. proliferatum* suggested sources for an exclusive metabolite that might be at least partially beneficial to the lifestyle specific to the fungus.

## 4. Conclusions

In summary, our study presented the high-quality genome sequence of the stain Fp9 of *F. proliferatum*, the causative agent of spikelet rot disease in rice. Despite the close phylogenetic relationships of *F. proliferatum* related to *F. fujikuroi,* there were sets of genes that were unique to *F. proliferatum* near the subtelomeric regions. The expansion of genes encoding MFS transporters and CWDEs was apparent in *F. proliferatum* compared with other fungi with different nutritional lifestyles, which seemed to be a specific feature of *F. proliferatum*. Although the high level of gene conservation and the collinearity of biosynthetic gene clusters responsible for mycotoxins was present among *F. proliferatum* and other fusaria, there were several differences in gene content and SM production, which provided the potential to contribute to the divergence of mycotoxins in the *Fusarium* species. To the best of our knowledge, this was the first whole-genome sequence of *F. proliferatum* isolated from rice. The genomic resources of the species foster the functional analysis of molecular components involved in pathogenicity, its interactions with host plants, and mycotoxins biosynthesis.

## 5. Materials and Methods

### 5.1. Fungal Strains and Culture Conditions

The *Fusarium proliferatum* strain Fp9 was isolated from naturally infected rice spikelets collected in Hangzhou, Zhejiang province, China [59]. Pathogenicity testing showed that the strain Fp9 caused the typical symptoms of spikelet rot in rice by artificial inoculation [59]. The strain was cultured in potato dextrose broth (PDB) for 5 to 7 days at 28 °C on an orbital shaker at 120 rpm, and mycelia were harvested by filtration. The strain was grown in YEPD medium (0.5% yeast extract, 1% peptone, and 2% glucose) to obtain spores, and stored in 20% glycerol stocks at −80 °C for long-term storage.

### 5.2. Species Identification

Genomic DNA was extracted from the mycelia using the DNeasy Plant Mini Kit (Qiagen, Hilden, Germany) according to the manufacturer’s instructions. The species was identified based on colony morphology and the PCR amplification of three genes encoding internal transcribed spacer (*ITS*) region, translation elongation factor (*TEF*), and beta-tubulin (*β-TUB*) using the primer pairs ITS1/ITS4 [60], EF1/EF2 [61], and T1/T22 [62], respectively. The PCR cycling parameters were as follows: an initial denaturation step at 94 °C for 5 min, 30 cycles at 94 °C for 15 s, 55 °C for 30 s, and 72 °C for 1 min, followed by a final extension step at 72 °C for 10 min. Each PCR reaction yielded a single band detected in a 1.0% (*w*/*v*) agarose gel stained with GelRed. PCR products were purified using the Wizard^®^ SV Gel and PCR Clean-Up System kit (Promega, Madison, WI, USA) and were directly sequenced in both directions using Sanger dideoxy chain termination in an ABI 3730 DNA sequencer at Sangon Company (Shanghai, China). A phylogeny tree was constructed using the neighbor-joining method implemented in MEGA [63] based on the concatenated alignment of the nucleotide sequences of genes with 1000 bootstrap replicates.

### 5.3. Genome Sequencing and De Novo Assembly

The genome was sequenced with massively parallel sequencing (MPS) Illumina technology. A paired-end library with 500-bp insert and two mate-pair libraries with 2-kb and 5-kb inserts were constructed and sequenced using an Illumina HiSeq2500 with PE125 strategy. Library construction and sequencing were performed at the Beijing Novogene Bioinformatics Technology Co., Ltd. (Beijing, China). The quality control of both paired-end and mate-pair reads was performed with an in-house program. Raw sequencing data were obtained through quality-filtering by removing the adapters and low-quality reads. The filtered reads were assembled into scaffolds by the software SOAPdenovo v2, Ruibang Luo, Hong Kong, China [64]. Genome size was evaluated based on Illumina short reads using the K-mer analysis toolkit [65]. The completeness of the genome assembly was assessed using BUSCO (benchmarking universal single copy ortholog) v5.2.2 with parameters ‘-m OGS’ [66].

### 5.4. Gene Prediction, Annotation and Protein Classification

Protein-encoding genes were predicted by the GeneMarkS programs [67]. Gene annotations were performed with BLAST search (*E*-value ≤ 10^−5^, minimal alignment length percentage ≥ 40%) against the following databases: NR [68], KEGG [69], COG [70], GO [71], Swiss-Prot, and TrEMBL databases [72]. Protein domains were defined in the Pfam [73] and Interpro [74] databases. Potential virulence-related proteins were identified by searching against the PHI-base [75]. Potential secreted proteins with signal peptide were predicted by SignalP 5.0 [76]. Membrane transporters were predicted by Phobius [77] and TMHMM [78], then classified based on the Transporter Classification Database [79]. The CAZymes were classified into different sub-families with the CAZyme database [80]. Biosynthetic gene clusters of SMs were analyzed using antiSMURF 4.0 with the Hidden Markov Model [81].

### 5.5. Orthology and Phylogenomic Analysis

To evaluate the phylogenomic position of *F. proliferatum*, the genomes of the other fungi were retrieved from NCBI database (Table S6). These fungi included the *Fusarium* species, *F. proliferatum* (*Gibberella* *fujikuroi* MP-D), *F. fujikuroi* (MP-C), *F. circinatum* (MP-H), *F. verticillioides* (MP-A), *F. graminearum* (non-GFC species), rice important fungal pathogens, *Magnaporthe oryzae*, *Ustilaginoidea virens*, *Rhizoctonia solani* AG-I IA, two model fungal species, *Neurospora crassa*, and *Saccharomyces cerevisiae*. Core/Pan genes were clustered by the CD-HIT [82] rapid clustering of similar proteins with a threshold of 50% pairwise identity and 0.7 length difference cutoff in amino acids. A genome-scale phylogenetic tree was constructed by the program TreeBeST using the maximum likelihood method of PhyML v3.0 [83], and the setting of bootstraps was 1000 calculated based on the protein sequences of the orthologous genes. Synteny analysis was performed using MUMmer v3.0 [84] package with a cluster length of exact matches of at least 100 nt and, at most, 500 nt mismatches between two exact matches.

### 5.6. Enrichment Analysis

Functional enrichment analysis was performed using the MIPS Functional Catalogue (FunCat) program [85]. FunCat categories with a false discovery rate (FDR) under 0.01 were defined as significantly enriched. The FDR correction of each annotated category was calculated using the Benjamini–Hochberg method. The enrichment analysis of gene families was conducted using the Gene Ontology Enrichment tools proposed online by the Eukaryotic Pathogen genome resource (EuPathDB) project [86] using biological ontology and InterPro predictions. The chi-squared test was used to compare the difference of the number of genes in the genome of *F. proliferatum* with the number of genes in the genome of other fungi. Over and underrepresentation were accepted at the significance threshold of *p*-value < 0.001.

### 5.7. Comparison of the Biosynthetic Gene Clusters of SMs

The content and arrangement of the biosynthetic gene cluster of SMs in *F. proliferatum* were compared to publicly available data and homologous sequences in other *Fusarium* species (Table 1). The genomes of *Fusarium* species examined were obtained from NCBI database (Table S6). The ortholog data of the biosynthetic gene cluster were used to determine the level of collinearity among *Fusarium* species. A fumonisin gene (*FUM*) cluster had been described in *F. verticillioides* [44]. A fusaric acid gene (*FUB*) cluster had been reported in *F. verticillioides* [87]. A bikaverin gene (*Bik*) cluster was characterized in *F. fujikuroi* [88]. A fusarubin gene (*FSR*) cluster was previously identified in *F. fujikuroi* [89]. An apicidin F gene (*APF*) cluster was characterized in *F. fujikuroi* [51]. A fusarin C gene (*FUS*) cluster was characterized in *F. fujikuroi* [53]. A gibberellins (GAs) gene cluster had been demonstrated in *F. fujikuroi* [90].

## Figures and Tables

**Table 1:** Descriptions of genes coding key biosynthetic enzymes for secondary metabolites (SMs) in *Fusarium* species.

Species	Secondary Metabolite	Gene	Predicted Function
*Fusarium verticillioides*	fumonisin	*FUM21*	Zn(II)2Cys6-type transcription factor
		*FUM1*	polyketide synthase
		*FUM6*	cytochrome P450 monooxygenase
		*FUM7*	dehydrogenase
		*FUM8*	aminotransferase
		*FUM3*	dioxygenase
		*FUM10*	fatty acyl-CoA synthetase
		*FUM11*	tricarboxylate transporter
		*FUM2*	cytochrome P450 monooxygenase
		*FUM20*	unknown
		*FUM13*	short-chain dehydrogenase/reductase
		*FUM14*	peptide synthetase
		*FUM15*	cytochrome P450 monooxygenase
		*FUM16*	fatty acyl-CoA synthetase
		*FUM17*	longevity assurance factor
		*FUM18*	longevity assurance factor
		*FUM19*	ABC transporter
*Fusarium verticillioides*	fusaric acid	*FUB1*	polyketide synthase
		*FUB2*	hypothetical protein of unknown function
		*FUB3*	amino acid kinase
		*FUB4*	hydrolase
		*FUB5*	acetyltransferase
		*FUB6*	dehydrogenase
		*FUB7*	sulfhydrylase
		*FUB8*	dehydrogenase
		*FUB9*	oxidase
		*FUB10*	C6 transcription factor
		*FUB11*	major facilitator superfamily transporter
		*FUB12*	C6 transcription factor
*Fusarium fujikuroi*	bikaverin	*Bik1*	polyketide synthase
		*Bik2*	putative FAD-dependent monooxygenase
		*Bik3*	O-methyltransferase
		*Bik4*	putative NmrA-like transcriptional regulator
		*Bik5*	Zn(II)2Cys6-type transcription factor
		*Bik6*	major facilitator superfamily transporter
*Fusarium fujikuroi*	fusarubin	*FSR1*	β-ketoacyl synthase
		*FSR2*	O-methyltransferase
		*FSR3*	monooxygenase
		*FSR4*	alcohol dehydrogenase
		*FSR5*	short-chain dehydrogenase/reductase
		*FSR6*	Zn(II)2Cys6-type transcription factor
*Fusarium fujikuroi*	apicidin	*APF1*	non-ribosomal peptide synthetase
		*APF2*	transcription factor
		*APF3*	Δ1-Pyrroline-5-carboxylate reductase
		*APF4*	aminotransferase
		*APF5*	fatty acid synthase
		*APF6*	O-methyltransferase
		*APF7*	cytochrome P450 monooxygenase
		*APF8*	cytochrome P450 monooxygenase
		*APF9*	FAD-dependent monooxygenase
		*APF11*	major facilitator superfamily transporter
		*APF12*	cytochrome b5-like
*Fusarium fujikuroi*	fusarin C	*FUS1*	polyketide synthase/nonribosomal peptide synthetase
		*FUS2*	α/β hydrolase
		*FUS3*	glutathione S-transferase
		*FUS4*	peptidase
		*FUS5*	serine hydrolase
		*FUS6*	MFS transporter
		*FUS7*	aldehyde dehydrogenase
		*FUS8*	cytochrome P450 monooxygenase
		*FUS9*	methyltransferase
*Fusarium fujikuroi*	gibberellins	*GGS2*	geranylgeranyl diphosphate synthase
		*DES*	GA4 desaturase
		*CPS/KS*	bifunctional ent-copalyl diphosphate/ent-kaurene synthase
		*P450-1*	cytochrome P450 monooxygenase
		*P450-2*	cytochrome P450 monooxygenase
		*P450-3*	cytochrome P450 monooxygenase
		*P450-4*	cytochrome P450 monooxygenase

## Data Availability

Not applicable.

## Supplementary Materials

The following supporting information can be downloaded at: https://www.mdpi.com/article/10.3390/toxins14080568/s1, Figure S1: Neighbor-joining tree inferred from the concatenated nucleotide sequences of *ITS*, *TEF-1α* and *β-TUB* of the strains of *F. proliferatum* and other *Fusarium* species; Figure S2: Gel electrophoresis images of PCR validation of structural variants (SVs) between *F. proliferatum* and *F. fujikuroi*; Table S1: Specific regions in the subtelomeres are present in *F. proliferatum* but absent in *F. fujikuroi*; Table S2: GO enrichment analysis of genes are present in the genome of *F. proliferatum* and absent in that of *F. fujikuroi*; Table S3: Summary of PCR validations of structural variants (SVs) between *F. proliferatum* and *F. fujikuroi*. Table S4: Comparison of genes coding for membrane transporters in *F. proliferatum* with that in other fungi; Table S5: Characterization of carbohydrate-active enzymes (CAZymes) of *F. proliferatum* and other fungi; Table S6: The protein-encoding genes and genomes of species used in this study; Additional file S1: Sanger sequencing results of structural variants (SVs) based on PCR validation for *F. proliferatum* and *F. fujikuroi*.
