# Supplementary material for: Genome-Wide Characterization Reveals Variation Potentially Involved in Pathogenicity and Mycotoxins Biosynthesis of Fusarium proliferatum Causing Spikelet Rot Disease in Rice
Source: Toxins (Basel). 2022 Aug 19;14(8):568. doi: 10.3390/toxins14080568 (PMC9414198; doi:10.3390/toxins14080568)
Supplement: Supplementary file 1 [file toxins-14-00568-s001.zip › toxins-1848341-supplementary.pdf]

Supplementary Materials

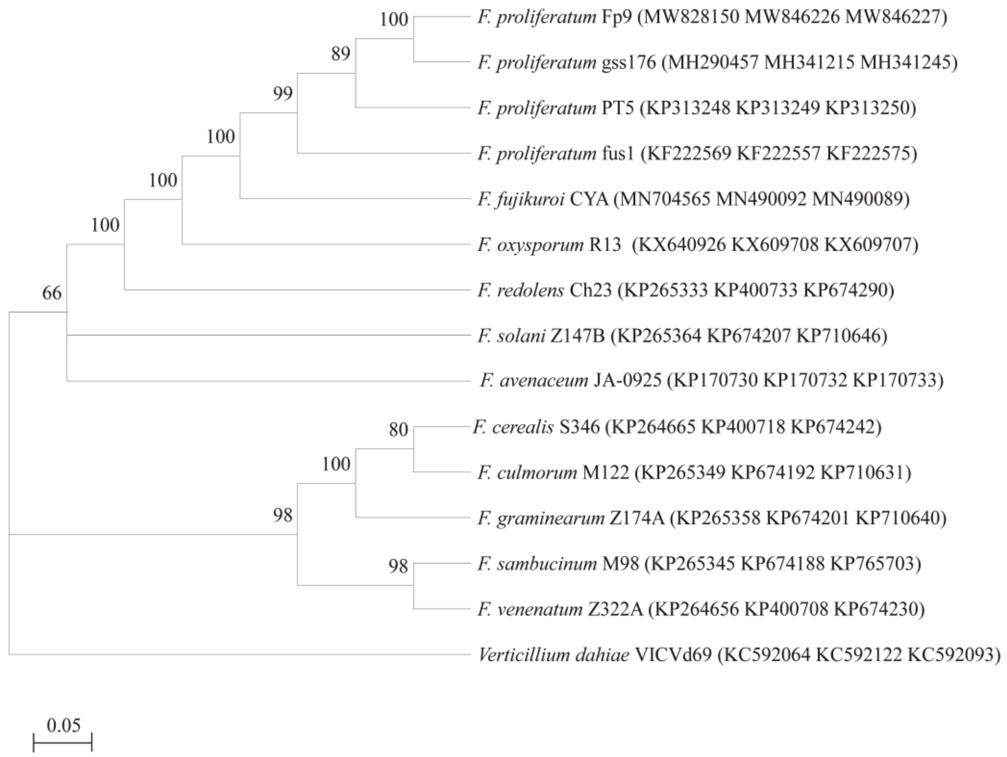

Figure S1. Neighbor-joining tree inferred from the concatenated nucleotide sequences of *ITS*, *TEF-1α* and *β-TUB* of the strains of *F. proliferatum* and other *Fusarium* species.

Note: *Verticillium dahiae* was used as an outgroup. The GenBank accession numbers from left to right in parentheses were the sequences of *ITS*, *TEF* and *β-TUB* genes, respectively. Numbers near branches were bootstrap values based on 1,000 replicates.

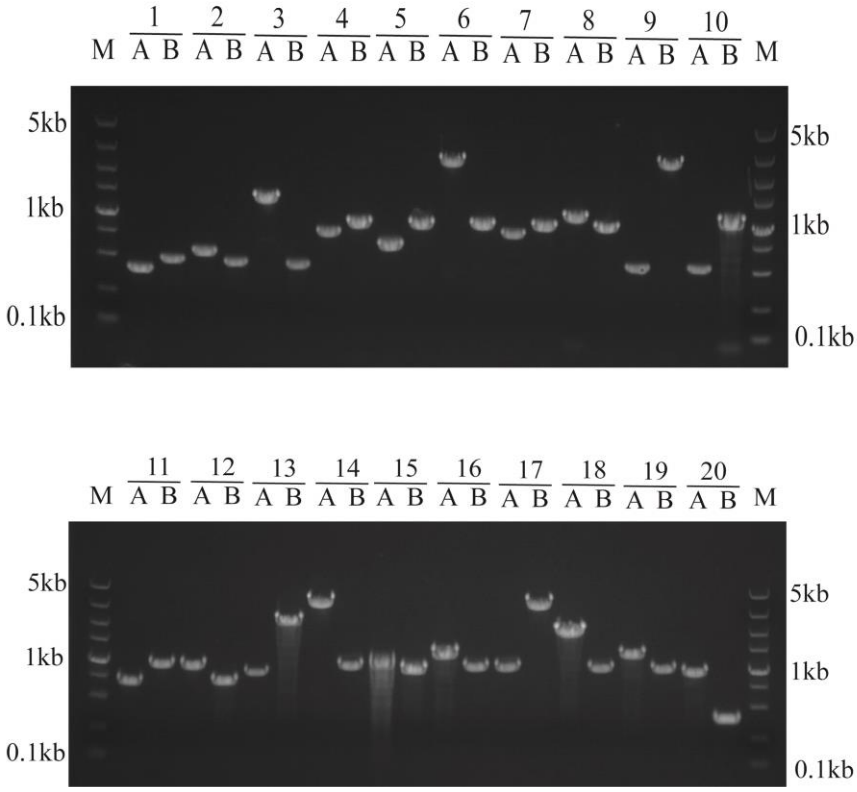

Figure S2. Gel electrophoresis images of PCR validation of structural variants (SVs) between *F. proliferatum* and *F. fujikuroi*

Note: PCR reactions used DNA from the *F. proliferatum* (A) and *F. fujikuroi* (B). Gel electrophoresis images of PCR products corresponding to the SVs in Table S3. Numbers above the small horizontal line represented SV ID. PCR validations of SV1 to SV10 was in top and SV11 to SV20 was in bottom, respectively.

Table S1 Specific regions in the subtelomeres present in *F. proliferatum* but absent in *F. fujikuroi*

Scaffold	Left/Right arms	Locus boundary	Fragments (bp)
Scaffold Ⅰ	Left	35777: 42225	6448
Scaffold Ⅰ	Right	6080517: 6114445	33928
Scaffold Ⅱ	Left	126954: 138359	11405
Scaffold Ⅱ	Right	4594735: 4601872	7137
Scaffold Ⅱ	Right	4729844: 4744065	14221
Scaffold Ⅳ	Left	14338: 70572	56234
Scaffold Ⅳ	Right	4454458: 4479090	24632
Scaffold Ⅶ	Right	2897101: 2974289	77188
Scaffold Ⅸ	Right	2876122: 2929835	53713
Scaffold Ⅸ	Right	2973049: 3023339	50290
Scaffold Ⅹ	Left	26521: 36495	9974
Scaffold Ⅹ	Right	79679: 91925	12246
Scaffold Ⅺ	Left	10360: 18871	8511
Scaffold Ⅺ	Right	339337: 364692	25355
Scaffold Ⅻ	Left	12623: 47357	34734
Scaffold Ⅻ	Right	619632: 674151	54519

Table S2 GO enrichment analysis of genes present in the genome of *F. proliferatum* and absent in that of *F. fujikuroi*

Type	GO ID	Function	No. of genes present in *F. proliferatum*	No. of genes present in *F. fujikuroi*
Biological Process	GO:0005975	carbohydrate metabolic process	1	5
	GO:0006139	nucleobase, nucleoside, nucleotide and nucleic acid metabolic process	1	0
	GO:0006200	ATP catabolic process	1	0
	GO:0006313	transposition, DNA-mediated	0	1
	GO:0006351	transcription, DNA-dependent	2	4
	GO:0006357	regulation of transcription from RNA polymerase II promoter	3	0
	GO:0006412	translation	1	0
	GO:0006418	tRNA aminoacylation for protein translation	1	0
	GO:0006457	protein folding	1	0
	GO:0006508	proteolysis	2	0
	GO:0006520	cellular amino acid metabolic process	0	1
	GO:0006629	lipid metabolic process	0	3
	GO:0006807	nitrogen compound metabolic process	2	0
	GO:0006950	response to stress	0	1
	GO:0007155	cell adhesion	0	1
	GO:0008152	metabolic process	0	5
	GO:0008643	carbohydrate transport	1	0
	GO:0009058	biosynthetic process	0	1
	GO:0009081	branched chain family amino acid metabolic process	1	0
	GO:0009098	leucine biosynthetic process	1	0
	GO:0009116	nucleoside metabolic process	1	3
	GO:0009409	response to cold	1	0
	GO:0009637	response to blue light	2	0
	GO:0009765	photosynthesis, light harvesting	1	0

	GO:0010114	response to red light	2	0
	GO:0010218	response to far red light	1	0
	GO:0015074	DNA integration	0	6
	GO:0016042	lipid catabolic process	0	2
	GO:0016310	phosphorylation	1	0
	GO:0018298	protein-chromophore linkage	1	0
	GO:0019253	reductive pentose-phosphate cycle	1	0
	GO:0031221	arabinan metabolic process	0	1
	GO:0044237	cellular metabolic process	1	0
	GO:0044249	cellular biosynthetic process	1	0
	GO:0045454	cell redox homeostasis	0	2
	GO:0046148	pigment biosynthetic process	1	0
	GO:0046373	L-arabinose metabolic process	0	1
	GO:0055085	transmembrane transport	3	8
	GO:0055114	oxidation-reduction process	7	0
	GO:0070941	eisosome assembly	0	11
	GO:0071704	organic substance metabolic process	1	0
**Cellular Component**	GO:0000124	SAGA complex	1	0
	GO:0005618	cell wall	1	0
	GO:0005623	cell	0	2
	GO:0005634	nucleus	1	9
	GO:0005737	cytoplasm	1	0
	GO:0005739	mitochondrion	1	0
	GO:0005832	chaperonin-containing T-complex	1	0
	GO:0009522	photosystem I	1	0
	GO:0009523	photosystem II	1	0
	GO:0009535	chloroplast thylakoid membrane	2	0
	GO:0009573	chloroplast ribulose bisphosphate carboxylase complex	1	0

	GO:0009941	chloroplast envelope	1	0
	GO:0010287	plastoglobule	1	0
	GO:0016020	membrane	1	0
	GO:0016021	integral to membrane	3	57
	GO:0022626	cytosolic ribosome	1	0
	GO:0031977	thylakoid lumen	1	0
	GO:0032126	eisosome	0	11
	GO:0048046	apoplast	3	0
**Molecular Function**	GO:0000166	nucleotide binding	4	0
	GO:0000287	magnesium ion binding	1	0
	GO:0000981	sequence-specific DNA binding RNA polymerase II transcription factor activity	3	8
	GO:0003676	nucleic acid binding	0	13
	GO:0003677	DNA binding	1	6
	GO:0003700	DNA-binding transcription factor activity	0	2
	GO:0003824	catalytic activity	0	5
	GO:0003862	3-isopropylmalate dehydrogenase activity	1	0
	GO:0003924	GTPase activity	0	1
	GO:0003979	UDP-glucose 6-dehydrogenase activity	1	0
	GO:0003995	acyl-CoA dehydrogenase activity	2	0
	GO:0004040	amidase activity	0	1
	GO:0004084	branched-chain-amino-acid transaminase activity	1	0
	GO:0004177	aminopeptidase activity	1	0
	GO:0004252	serine-type endopeptidase activity	1	2
	GO:0004386	helicase activity	0	1
	GO:0004402	histone acetyltransferase activity	0	1
	GO:0004497	monooxygenase activity	3	1
	GO:0004553	hydrolase activity, hydrolyzing O-glycosyl compounds	1	5

	GO:0004672	protein kinase activity	0	5
	GO:0004806	triglyceride lipase activity	0	1
	GO:0004812	aminoacyl-tRNA ligase activity	1	0
	GO:0005506	iron ion binding	1	1
	GO:0005507	copper ion binding	1	0
	GO:0005524	ATP binding	2	7
	GO:0005525	GTP binding	0	2
	GO:0008061	chitin binding	0	2
	GO:0008081	phosphoric diester hydrolase activity	0	2
	GO:0008171	O-methyltransferase activity	0	1
	GO:0008233	peptidase activity	1	0
	GO:0008236	serine-type peptidase activity	0	1
	GO:0008237	metallopeptidase activity	1	2
	GO:0008239	dipeptidyl-peptidase activity	1	0
	GO:0008270	zinc ion binding	1	9
	GO:0008408	3'-5' exonuclease activity	0	1
	GO:0008483	transaminase activity	1	1
	GO:0009055	electron transfer activity	0	1
	GO:0009853	photorespiration	1	0
	GO:0010218	response to far red light	1	0
	GO:0010484	H3 histone acetyltransferase activity	1	0
	GO:0016168	chlorophyll binding	1	0
	GO:0016301	kinase activity	2	0
	GO:0016407	acetyltransferase activity	0	1
	GO:0016491	oxidoreductase activity	4	7
	GO:0016614	oxidoreductase activity, acting on CH-OH group of donors	0	2
	GO:0016616	oxidoreductase activity, acting on the CH-OH group of donors, NAD or NADP as acceptor	0	1

	GO:0016620	oxidoreductase activity, acting on the aldehyde or oxo group of donors, NAD or NADP as acceptor	0	1
	GO:0016702	oxidoreductase activity, acting on single donors with incorporation of molecular oxygen, incorporation of two atoms of oxygen	0	1
	GO:0016705	oxidoreductase activity, acting on paired donors, with incorporation or reduction of molecular oxygen	1	1
	GO:0016747	transferase activity, transferring acyl groups other than amino-acyl groups	0	1
	GO:0016758	transferase activity, transferring hexosyl groups	1	0
	GO:0016772	transferase activity, transferring phosphorus-containing groups	1	0
	GO:0016787	hydrolase activity	1	7
	GO:0016798	hydrolase activity, acting on glycosyl bonds	1	0
	GO:0016810	hydrolase activity, acting on carbon-nitrogen (but not peptide) bonds	1	1
	GO:0016829	lyase activity	1	1
	GO:0016836	hydro-lyase activity	0	1
	GO:0016853	isomerase activity	0	2
	GO:0016984	ribulose-bisphosphate carboxylase activity	1	0
	GO:0020037	heme binding	1	1
	GO:0022857	transmembrane transporter activity	0	1
	GO:0022891	substrate-specific transmembrane transporter activity	1	0
	GO:0030151	molybdenum ion binding	0	1
	GO:0030170	pyridoxal phosphate binding	0	2
	GO:0030246	carbohydrate binding	0	1
	GO:0042626	ATPase activity, coupled to transmembrane movement of substances	1	0
	GO:0043169	cation binding	1	0
	GO:0043531	ADP binding	0	2
	GO:0046556	alpha-L-arabinofuranosidase activity	0	1
	GO:0046872	metal ion binding	4	3

	GO:0046873	metal ion transmembrane transporter activity	0	1
	GO:0050660	flavin adenine dinucleotide binding	1	2
	GO:0050662	coenzyme binding	1	0
	GO:0051082	unfolded protein binding	1	0
	GO:0051213	dioxygenase activity	0	1
	GO:0051287	NAD binding	2	0
	GO:0051537	2 iron, 2 sulfur cluster binding	0	1
	GO:0051920	peroxiredoxin activity	0	1
	GO:0052664	nitroalkane oxidase activity	1	0
	GO:0070011	peptidase activity, acting on L-amino acid peptides	1	0
	GO:0071949	FAD binding	1	2
	GO:0071704	organic substance metabolic process	1	0

Table S3 Summary of PCR validations of structural variants (SVs) between *F. proliferatum* and *F. fujikuroi*

SV ID	SV class	SV size (bp)	A (bp)	B (bp)	Forward Primer (5'-3')	Reverse Primer (5'-3')
1	Deletion	80	418	495	AGGGGACAAGAGGTAAG	TCAGGCTATCTGTGCAG
2	Insertion	101	610	509	TAACCTCTCCTATCTCT	ACGCTCCCTTGAACAAC
3	Insertion	892	1386	494	ACTCAGTCTGTCCGTTG	GGTCCTGTTTTCCTCTA
4	Deletion	162	840	1005	CGGATAGTCGTCTTCAT	AACTGATTTGAGAGCCC
5	Deletion	280	728	1008	ATGACAACCATGAATCA	AACAAGAAGACCCTGTG
6	Insertion	1476	2443	968	ATTTATGTGATGCCGCC	ATCGACAACTCATGCTTGA
7	Deletion	145	843	993	GCCTCGGGATATGGAGG	TCGAGAACAGGGATGAGG
8	Insertion	146	1107	959	GTCTCCTCTTTCCTCAC	GAACCAACAGAACCTTT
9	Deletion	1968	573	2541	AGGTCGGTGTCGGTATC	GCTTGTGTGCGGGTGTC
10	Deletion	579	591	1170	GCTGCCCATCATAAGTG	TCACCCCAATCTCGGT
11	Deletion	295	709	998	CTCTAATAGTGATGCGAGGTG	AGCGTTTTGTTTGGGTC
12	Insertion	206	973	767	TGCCCAGTGATAACGA	AGATGGAAAGAACATGATG
13	Deletion	1383	848	2231	ATGTCTTCAAATGTAACGAGA	TGTGAGCCAGCGGGTTA
14	Insertion	2008	2915	908	CCTCGGTTCTGCTGTCT	TGTTTGCTGTGAAGTCT
15	Insertion	153	1103	950	ACGGCGTTGGATATATA	TACCAATGGTTCTCAAT
16	Insertion	290	1261	982	ATGACTCTCGTGTTAAG	TTCCATTTATTCTTTGAA
17	Deletion	1850	985	2828	GTTTCGTCGCCAAAGCT	AACCATGAGGCTTCAGC
18	Insertion	1067	2017	950	GCGTGTTGACCCATTTC	CGCAACTTGATTTAGCC
19	Insertion	409	1407	998	AGTATCTGAACCAATGC	AGAAGGAACTAGTCGTG
20	Insertion	743	1105	362	AAAGCAGTGGCGAACAT	CAGCAAAACGAAAACGA

Note: A indicated PCR size (bp) in *F. proliferatum* strain Fp9; B indicated PCR size (bp) in *F. fujikuroi* strain IMI 58289.

Table S4 Comparison of genes encoding for membrane transporters in *F. proliferatum* with that in other fungi

Superfamilies	TC No.	Subclass description	FP	FG	MO	BC	SS	NC	AN
Channels/Pores	1.A	α-Type channels	69	76	64	64	118	52	52
	1.B	β-Barrel porins	1	1	2	0	2	1	0
	1.C	Pore-forming toxins(proteins and peptides)	6	4	5	7	12	6	4
	1.F	Vesicle Fusion Pores	5	8	6	3	7	4	6
	1.H	Paracellular channels	5	5	3	3	6	3	3
	1.I	Membrane-bounded Channels	10	8	5	3	7	5	4
	1.P	Non-Envelop Virus Penitration Complex: A complex of host cell proteins that allow non-envelop virus to penetrate the endoplasmic reticular membrane.	2	3	2	1	2	3	3
	1.Q	Fungal Septal Pores	1	1	1	0	1	1	1
Electrochemical potential-driven transporpters	2.A	Porters (uniporters, symporters, antiporters)	905	772	501	517	879	341	636
	2.A.1	The Major Facilitator Superfamily (MFS)	567	421	271	299	451	157	390
	2.A.2	The Glycoside-Pentoside-Hexuronide (GPH):Cation Symporter Family	2	2	4	4	6	2	2
	2.A.3	The Amino Acid-Polyamine-Organocation (APC) Superfamily	74	71	32	45	80	18	57
	2.A.4	The Cation Diffusion Facilitator (CDF) Family	9	7	8	6	13	9	6
	2.A.5	The Zinc (Zn^{2+})-Iron (Fe^{2+}) Permease (ZIP) Family	10	9	8	8	14	8	7
	2.A.6	The Resistance-Nodulation-Cell Division (RND) Superfamily	3	3	3	3	6	3	2
	2.A.7	The Drug/Metabolite Transporter (DMT) Superfamily	24	29	23	16	41	21	20
	2.A.9	The Membrane Protein Insertase (YidC/Alb3/Oxa1) Family	2	1	1	1	4	1	2
	2.A.16	The Tellurite-resistance/Dicarboxylate Transporter (TDT) Family	8	12	5	5	6	3	4
	2.A.17	The Proton-dependent Oligopeptide Transporter (POT) Family	6	5	4	3	9	2	4
	2.A.18	The Amino Acid/Auxin Permease (AAAP) Family	26	32	12	12	22	6	14
	2.A.19	The Ca^{2+}:Cation Antiporter (CaCA) Family	8	10	7	10	19	10	6
	2.A.20	The Inorganic Phosphate Transporter (PiT) Family	4	4	3	1	2	0	4
	2.A.21	The Solute:Sodium Symporter (SSS) Family	5	3	3	2	2	3	4
	2.A.22	The Neurotransmitter:Sodium Symporter (NSS) Family	2	3	3	0	0	0	1
	2.A.23	The Dicarboxylate/Amino Acid:Cation (Na^+ or H^+) Symporter (DAACS) Family	1	1	1	0	0	0	1

2.A.25	The Alanine or Glycine:Cation Symporter (AGCS) Family	0	0	1	0	1	0	0
2.A.28	The Bile Acid:Na^+ Symporter (BASS) Family	1	1	1	0	0	0	1
2.A.29	The Mitochondrial Carrier (MC) Family	17	16	13	14	23	16	17
2.A.30	The Cation-Chloride Cotransporter (CCC) Family	1	1	1	1	2	1	1
2.A.31	The Anion Exchanger (AE) Family	3	4	1	2	2	2	2
2.A.36	The Monovalent Cation:Proton Antiporter-1 (CPA1) Family	4	5	4	5	8	3	6
2.A.37	The Monovalent Cation:Proton Antiporter-2 (CPA2) Family	2	1	3	2	4	2	2
2.A.38	The K+ Transporter (Trk) Family	5	6	5	3	5	2	3
2.A.39	The Nucleobase:Cation Symporter-1 (NCS1) Family	13	9	7	8	17	3	11
2.A.40	The Nucleobase:Cation Symporter-2 (NCS2) Family	5	4	3	3	8	4	3
2.A.41	The Concentrative Nucleoside Transporter (CNT) Family	1	1	1	0	0	1	1
2.A.43	The Lysosomal Cystine Transporter (LCT) Family	6	6	7	5	12	7	5
2.A.47	The Divalent Anion:Na+ Symporter (DASS) Family	1	2	1	1	2	1	1
2.A.49	The Chloride Carrier/Channel (ClC) Family	4	5	3	3	7	4	3
2.A.50	The Glycerol Uptake (GUP) Family	1	1	1	1	2	2	1
2.A.51	The Chromate Ion Transporter (CHR) Family	1	2	1	0	0	1	2
2.A.52	The Ni^{2+}-Co^{2+} Transporter (NiCoT) Family	1	2	2	1	2	1	1
2.A.53	The Sulfate Permease (SulP) Family	7	8	8	4	10	4	4
2.A.54	The Mitochondrial Tricarboxylate Carrier (MTC) Family	1	1	1	1	2	1	1
2.A.55	The Metal Ion (Mn^{2+}-iron) Transporter (Nramp) Family	2	1	0	1	4	2	1
2.A.57	The Equilibrative Nucleoside Transporter (ENT) Family	3	4	2	2	4	1	2
2.A.59	The Arsenical Resistance-3 (ACR3) Family	2	1	1	1	2	1	1
2.A.66	The Multidrug/Oligosaccharidyl-lipid/Polysaccharide (MOP) Flippase Superfamily	6	8	4	3	7	5	5
2.A.67	The Oligopeptide Transporter (OPT) Family	13	13	12	12	23	11	6
2.A.69	The Auxin Efflux Carrier (AEC) Family	1	2	2	3	6	2	2
2.A.72	The K^+ Uptake Permease (KUP) Family	1	1	1	1	2	0	0
2.A.77	The Cadmium Resistance (CadD) Family	1	1	1	0	0	0	0

	2.A.79	The Threonine/Serine Exporter (ThrE) Family	2	2	1	1	2	1	3
	2.A.82	The Organic Solute Transporter (OST) Family	2	2	2	3	4	2	2
	2.A.85	The Aromatic Acid Exporter (ArAE) Family	6	7	2	5	8	2	4
	2.A.89	The Vacuolar Iron Transporter (VIT) Family	2	2	1	1	2	1	2
	2.A.90	The Vitamin A Receptor/Transporter (STRA6) Family	1	1	1	1	2	1	1
	2.A.92	The Choline Transporter-like (CTL) Family	1	1	1	0	1		1
	2.A.94	The Phosphate Permease (Pho1) Family	2	2	3	2	4	3	2
	2.A.96	The YaaH (YaaH) Family	11	7	2	3	4	1	6
	2.A.97	The Mitochondrial Inner Membrane K^+/H^+ and Ca^{2+}/H^+ Exchanger (LetM1) Family	0	0	1	1	2	1	1
	2.A.100	The Ferroportin (Fpn) Family	1	2	1	1	2	1	1
	2.A.105	The Mitochondrial Pyruvate Carrier (MPC) Family	1	1	3	1	2	2	1
	2.A.106	The Ca^{2+}:H^+ Antiporter-2 (CaCA2) Family	1	2	1	1	2	1	1
	2.A.108	The Iron/Lead Transporter (ILT) Family	9	9	4	3	6	2	
	2.A.121	The Sulfate Transporter (CysZ) Family	2	3	3	1	4	2	2
	2.A.122	The LrgB/CidB Holin-like Glycolate/Glycerate Transporter (LrgB/CidB/GGT) Family	2	2	0	0	2	0	0
	2.A.126	The Fatty Acid Exporter (FAX) Family	1	2	0	1	2	1	2
	2.A.127	The Enterobacterial Cardiolipin Transporter (CLT) Family	7	8	0	0	0	0	1
Primary active transporters	3.A	P–P-bond-hydrolysis-driven transporters	127	147	111	105	187	75	99
	3.A.1	The ATP-binding Cassette (ABC) Superfamily	59	65	42	47	79	27	45
	3.A.2	The H^+- or Na^+-translocating F-type, V-type and A-type ATPase (F-ATPase) Superfamily	7	12	8	6	14	3	6
	3.A.3	The P-type ATPase (P-ATPase) Superfamily	30	32	24	20	33	19	21
	3.A.4	The Arsenite-Antimonite (ArsAB) Efflux Family	1	1	1	0	2	0	0
	3.A.5	The General Secretory Pathway (Sec) Family	8	7	8	6	14	5	7
	3.A.8	The Mitochondrial Protein Translocase (MPT) Family	5	5	5	8	12	1	6
	3.A.9	The Chloroplast Envelope Protein Translocase (CEPT or Tic-Toc) Family	0	3	3	1	0	0	0
	3.A.16	The Endoplasmic Reticular Retrotranslocon (ER-RT) Fam	6	8	8	4	10	6	5

	3.A.19	The TMS Recognition/Insertion Complex (TRC) Family	1	1	0	1	1		1
	3.A.20	The Peroxisomal Protein Importer (PPI) Family	2	4	2	4	6	5	2
	3.A.25	The Symbiont-specific ERAD-like Machinery (SELMA) Family	1	0	1	0	1	1	1
	3.A.27	The C-terminal Tail-Anchored Membrane Protein Biogenesis/ Insertion Complex-2 (TAMP-B2) Family	4	4	6	4	8	4	3
	3.A.28	The AAA-ATPase, Bcs1 (Bcs1) Family	2	3	2	3	5	2	2
	3.A.29	The Mitochondrial Inner Membrane i-AAA Protease Complex (MIMP) Familly	1	2	1	1	2	2	0
	3.B	Decarboxylation-driven transporters	0	0	0	0	0	0	1
	3.D	Oxidoreduction-driven transporters	10	27	10	8	34	9	11
	3.E	Light absorption-driven transporters	5	4	1	2	4	1	1
	4.C	Acyl CoA ligase-coupled transporters	11	8	3	3	3	4	4
	4.D	Polysaccharide Synthase/Exporters	27	31	14	21	44	14	14
	4.E	Vacuolar Polyphosphate Polymerase-catalyzed Group Translocators	2	4	2	2	4	2	1
	4.F	Choline/EthanolaminePhosphotransferase 1 (CEPT1)	3	4	4	3	6	4	3
Transmembrane electron carriers	5.B	Transmembrane one-electron transfer carriers	26	34	20	19	31	15	15
Accessory electron carriers	8.A	Auxiliary transport proteins	29	38	26	24	56	28	25
	8.B	Ribosomally synthesized protein/peptide toxins that target channels and carriers	0	1	1	0	0	0	0
Incompletely characterized transporters	9.A	Recognized transporters of unknown biochemical mechanism	74	70	29	42	58	27	46
	9.B	Putative uncharacterized transport proteins	84	103	87	65	164	75	81
**Total**			**1402**	**1349**	**897**	**892**	**162 5**	**670**	**1010**

Note: Organisms, FP, *Fusarium proliferatum*; FG, *Fusarium graminearum*; MO, *Magnaporthe oryzae*; BC, *Botrytis cinerea*; SS, *Sclerotinia sclerotiorum*; NC, *Neuropora crassa*; AN, *Aspergillus nidulans*.

Table S5 Characterization of carbohydrate-active enzymes (CAZymes) of *F. proliferatum* and other fungi

Species	Glycoside Hydrolase Family (GHs)																								
	**1**	**2**	**3**	**5**	**6**	**7**	**8**	**9**	**10**	**11**	**12**	**13**	**15**	**16**	**17**	**18**	**20**	**23**	**24**	**25**	**26**	**27**	**28**	**29**	**30**	**31**
FP	5	10	25	19	1	3	0	0	5	3	4	9	4	24	6	18	4	0	1	0	0	4	12	3	3	8
FG	3	10	21	14	1	2	0	0	5	3	4	8	3	24	6	19	3	0	0	0	0	2	6	1	0	8
FF	4	9	24	18	1	3	0	0	3	3	4	10	3	24	6	17	4	0	1	0	0	3	8	2	2	8
LM	3	7	13	15	3	3	0	0	3	2	3	7	3	18	8	11	2	0	1	0	0	4	7	1	2	8
MO	2	8	18	13	3	6	0	0	6	5	3	11	2	18	7	17	3	0	1	0	0	4	4	4	1	6
BC	3	2	16	16	1	2	0	0	2	3	5	11	5	21	6	11	1	0	0	0	2	4	19	0	0	4
SS	3	2	13	14	1	3	0	0	2	3	5	10	4	20	6	14	1	0	0	1	1	4	17	0	0	6
AN	3	10	20	15	2	3	0	0	3	2	1	14	2	13	5	9	2	0	1	3	3	3	9	0	0	10
NC	1	5	9	7	3	5	0	0	4	2	1	10	2	14	4	12	1	0	0	1	1	0	2	0	2	5
UB	0	2	4	10	0	0	1	1	2	1	0	5	1	14	2	2	2	1	0	1	1	1	1	0	2	3
MP	0	1	2	10	0	0	0	1	1	1	0	4	1	16	2	3	2	1	0	0	2	3	1	0	2	3

Species	Glycoside Hydrolase Family (GHs)																								
	**32**	**33**	**35**	**36**	**37**	**38**	**39**	**42**	**43**	**45**	**47**	**49**	**51**	**53**	**54**	**55**	**62**	**63**	**64**	**65**	**67**	**71**	**72**	**74**	**75**	**76**
FP	7	1	3	4	2	1	3	0	27	1	10	2	2	1	1	2	1	1	2	1	2	3	4	1	3	9
FG	5	1	3	3	2	1	2	0	18	1	10	0	2	1	1	0	2	1	2	0	1	0	3	1	1	8
FF	6	1	2	4	2	1	5	0	27	1	10	1	2	1	2	0	1	1	2	1	2	2	3	0	2	9
LM	2	4	4	2	2	1	1	0	12	2	8	0	3	1	0	0	1	3	1	1	1	1	6	0	0	7
MO	5	0	0	2	2	2	2	0	19	1	9	0	3	1	1	0	4	2	2	0	1	1	5	1	1	8
BC	1	0	4	2	1	1	1	0	5	2	8	0	4	2	1	0	1	1	2	2	0	11	6	0	0	11
SS	1	0	4	2	1	1	0	0	5	2	8	0	2	2	1	0	0	1	2	3	0	10	6	0	0	11
AN	2	0	4	4	1	1	1	0	18	1	7	0	2	1	1	6	2	1	0	1	1	5	5	2	2	7
NC	0	0	2	1	2	1	0	0	7	1	8	0	1	1	1	0	0	1	2	0	1	6	5	1	1	10
UB	3	0	1	1	2	1	0	1	3	4	3	0	3	0	1	0	1	1	0	0	0	0	1	0	0	0

MP	2	0	1	1	1	1	0	1	5	2	4	1	1	0	1	0	0	1	0	0	0	0	1	0	0	0

**Species**	**Glycoside Hydrolase Family (GHs)**																									
	**78**	**79**	**81**	**85**	**88**	**89**	**92**	**93**	**94**	**95**	**105**	**114**	**115**	**125**	**127**	**128**	**131**	**132**	**133**	**134**	**135**	**141**	**142**	**145**	**146**	**No classified**	**Total**
FP	12	1	3	0	3	0	0	3	0	1	3	3	2	3	1	3	1	3	1	0	0	0	0	0	0	0	**308**
FG	7	1	1	0	1	0	0	2	0	2	3	2	2	3	1	4	1	2	1	0	0	0	1	1	1	4	**252**
FF	9	2	3	0	3	0	0	4	0	2	4	2	2	3	2	4	1	2	1	1	0	0	1	1	1	6	**299**
LM	1	1	2	1	1	0	6	2	0	1	3	2	2	3	0	4	2	1	1	1	0	1	0	0	1	4	**227**
MO	3	2	2	0	1	0	6	1	1	1	4	1	3	6	0	5	6	2	2	0	0	0	0	1	2	2	**265**
BC	8	2	1	0	1	0	5	1	0	2	1	1	1	3	1	1	1	2	1	0	2	0	0	1	0	0	**235**
SS	4	2	1	0	0	1	5	1	0	1	1	1	1	3	0	3	2	2	1	0	3	0	0	0	0	9	**233**
AN	8	1	2	0	2	0	5	2	0	3	3	2	1	1	1	2	1	2	2	4	3	0	0	0	0	4	**257**
NC	0	2	1	0	0	0	2	2	1	0	1	1	1	3	0	2	3	2	1	0	0	0	2	1	0	2	**170**
UB	0	1	0	1	0	0	3	1	0	0	1	0	1	0	0	5	0	0	1	0	1	0	0	0	0	3	**101**
MP	0	1	0	1	0	0	2	1	0	0	1	0	1	1	0	5	0	0	1	0	0	0	0	0	0	5	**98**

Species	**Glycosyltransferase Family (GTs)**																	
	1	2	3	4	5	8	15	17	20	21	22	24	25	30	31	32	33	34
FP	18	1	1	0	0	7	5	2	3	1	4	1	0	1	7	5	1	3
FG	16	20	1	0	0	6	4	2	3	1	4	1	0	0	5	5	1	2
FF	13	23	1	0	0	7	5	2	3	1	4	1	0	0	6	6	1	3
LM	6	16	1	0	0	9	3	0	3	1	4	1	2	0	7	5	1	7
MO	13	11	1	0	1	2	4	1	3	1	4	1	6	0	4	10	1	2
BC	13	13	1	0	1	7	4	2	3	1	4	1	2	0	6	6	0	1
SS	10	17	1	0	1	6	4	2	3	1	4	1	3	0	6	4	1	2

AN	9	12	1	7	2	4	3	0	3	1	4	1	4	0	5	7	1	3
NC	6	11	1	0	2	2	5	1	3	1	3	1	1	0	3	4	1	2
UB	5	15	1	0	0	2	1	1	3	1	4	1	0	0	3	2	1	0
MP	5	13	1	0	0	2	2	0	3	1	3	1	0	0	3	2	1	0

**Species**	**Glycosyltransferase Family (GTs)**																	
	**35**	**39**	**41**	**48**	**50**	**55**	**57**	**58**	**59**	**61**	**62**	**64**	**66**	**69**	**71**	**76**	**90**	**No classified**	**Total**
FP	1	3	1	1	1	0	2	1	1	0	3	2	1	3	3	1	5	0	**89**
FG	1	3	1	1	1	0	2	1	1	0	3	2	1	2	4	1	6	4	**105**
FF	1	3	1	1	1	0	2	1	1	0	3	2	1	3	3	1	6	2	**108**
LM	1	3	1	1	1	0	2	1	1	0	3	0	1	2	2	1	4	2	**92**
MO	1	3	1	1	1	1	2	1	1	3	3	0	1	4	4	1	6	2	**101**
BC	1	3	1	2	1	0	2	1	0	0	3	0	1	3	5	2	3	6	**99**
SS	1	3	1	1	1	0	2	1	1	0	3	0	1	2	2	1	3	4	**93**
AN	1	3	1	1	1	0	2	1	1	0	3	0	1	3	2	0	2	2	**91**
NC	1	3	1	1	1	1	2	1	1	0	3	0	1	2	1	1	3	4	**74**
UB	0	3	1	1	1	0	2	1	1	0	0	0	1	2	2	1	1	0	**57**
MP	0	3	1	1	1	1	2	1	0	0	0	0	1	1	2	1	2	3	**57**

**Species**	**Polysaccharide Lyase Family (PLs)**									
	**1**	**3**	**4**	**9**	**11**	**14**	**20**	**26**	**27**	**No classified**	**Total**
FP	11	7	3	2	0	0	0	0	0	0	**23**
FG	9	7	3	1	0	0	1	1	0	0	**22**
FF	11	7	2	2	0	0	0	1	0	0	**23**

LM	9	6	4	1	0	0	0	1	0	0	**21**
MO	2	1	1	0	0	0	1	1	0	0	**6**
BC	6	2	0	0	0	0	0	0	0	0	**8**
SS	4	0	0	0	0	0	0	0	0	0	**4**
AN	8	5	4	1	1	0	2	1	1	0	**23**
NC	1	1	1	0	0	0	1	0	0	0	**4**
UB	1	0	0	0	0	1	0	0	0	1	**3**
MP	1	0	0	0	0	1	0	0	0	1	**3**

**Species**	**Carbohydrate Esterase Family (CEs)**										
	**1**	**2**	**3**	**4**	**5**	**8**	**9**	**12**	**15**	**16**	**No classified**	**Total**
FP	7	1	9	8	11	4	1	3	0	5	0	**49**
FG	4	1	5	7	13	6	1	3	0	5	0	**45**
FF	3	1	7	9	11	7	1	3	0	5	0	**47**
LM	3	1	2	7	8	3	2	3	2	2	0	**33**
MO	10	1	6	9	17	1	1	3	1	2	0	**51**
BC	2	0	1	4	11	5	2	3	0	6	1	**35**
SS	2	0	1	4	8	5	2	3	1	6	1	**33**
AN	1	0	5	6	4	3	1	2	0	3	4	**29**
NC	6	0	3	3	3	1	1	1	1	1	1	**21**
UB	1	0	0	5	2	1	1	0	0	0	0	**10**
MP	0	0	0	7	1	1	1	0	0	0	0	**10**

Species	Auxiliary Activity Family (AAs)													
	**1**	**2**	**3**	**4**	**5**	**6**	**7**	**8**	**9**	**10**	**11**	**12**	**13**	**Total**
FP	14	3	29	0	5	2	5	7	14	0	4	3	0	**86**
FG	14	1	20	0	5	1	4	4	13	0	4	2	1	**69**
FF	12	0	24	0	4	2	4	3	13	0	4	1	1	**68**
LM	2	0	4	0	2	0	1	3	20	0	3	3	1	**39**
MO	15	3	22	0	4	1	3	7	24	0	7	5	1	**92**
BC	1	0	6	0	3	1	1	3	9	0	2	0	1	**27**
SS	8	0	23	0	5	1	1	2	9	0	2	1	1	**53**
AN	2	0	16	0	0	0	0	2	9	0	2	0	2	**33**
NC	8	0	2	0	1	0	0	2	14	0	4	3	1	**35**
UB	5	0	7	0	2	1	2	0	0	1	0	0	0	**18**
MP	5	0	8	0	3	1	2	0	0	1	0	0	0	**20**

Species	Carbohydrate-Binding Module Family (CBMs)																				
	**1**	**2**	**5**	**6**	**13**	**14**	**18**	**20**	**21**	**24**	**32**	**35**	**38**	**42**	**43**	**48**	**50**	**52**	**63**	**66**	**No classified**	**Total**
FP	14	1	2	1	12	0	11	5	1	1	3	4	1	1	1	3	2	0	4	0	0	**67**
FG	13	0	0	1	2	0	34	2	2	0	3	3	0	1	1	3	19	0	3	0	0	**87**
FF	12	0	0	1	1	0	22	2	2	2	3	3	1	2	1	3	21	0	3	0	0	**79**
LM	5	0	0	0	0	0	30	3	1	0	1	4	0	0	2	2	5	0	1	0	1	**55**
MO	28	0	0	2	0	0	53	3	1	0	0	3	0	2	2	3	14	1	1	1	0	**114**
BC	17	0	0	0	1	0	17	3	1	17	2	3	0	1	1	3	1	0	1	0	0	**68**
SS	24	0	0	0	1	0	28	3	1	17	2	2	0	1	1	3	10	0	1	0	0	**94**
AN	7	0	0	1	0	1	17	4	1	2	0	3	0	1	1	1	4	0	1	0	0	**44**
NC	19	0	0	0	2	0	3	2	1	8	1	0	1	0	1	1	2	1	0	0	0	**42**

UB	0	0	0	0	0	0	1	0	1	0	0	1	0	0	1	2	0	0	0	0	0	**6**
MP	0	0	0	0	0	0	1	0	1	0	0	1	0	0	1	2	0	0	0	0	0	**6**

Note: The enzyme classes were predicted by the CAZyme database (http://www.cazy.org/). Organisms, FP, *Fusarium proliferatum*; FG, *Fusarium graminearum*; FF, *Fusarium fujikuroi*; LM, *Leptosphaeria maculans*; MO, *Magnaporthe oryzae*; BC, *Botrytis cinerea*; SS, *Sclerotinia sclerotiorum*; AN, *Aspergillus nidulans*; NC, *Neurospora crassa*; UB, *Ustilago bromivora*; MP, *Melanopsichium pennsylvanicum*.

Table S6 The protein-encoding genes and genomes of species used in this study

Species	Genome size (Mb)	No. of genes	NCBI accessions	BioProject	BioSample
Aspergillus nidulans	30.05	10,045	AACD00000000	PRJNA130	SAMN02953587
Aspergillus niger	34.85	10,947	ACJE00000000	PRJNA15785	SAMN02953752
Botrytis cinerea	42.63	13,703	AAID00000000	PRJNA15632	SAMN02953628
Fusarium circinatum	43.95	15,022	AYJV00000000	PRJNA41113	SAMN02371877
Fusarium fujikuroi	43.83	14,810	HF679023 to HF679034	PRJNA322155	SAMEA3724789
Fusarium graminearum	36.45	13,826	AACM00000000	PRJNA13839	SAMN02953593
Fusarium oxysporum	61.38	17,458	AAXH00000000	PRJNA18813	SAMN02953675
Fusarium solani	45.81	15,702	NGZQ00000000	PRJNA368786	SAMN06272960
Fusarium verticillioides	41.84	14,180	AAIM00000000	PRJNA15553	SAMN02953630
Leptosphaeria maculans	45.12	12,469	UNSE01000000	PRJEB24468	SAMEA104483101
Magnaporthe oryzae	40.97	13,184	AACU00000000	PRJNA13840	SAMN02953596
Melanopsichium pennsylvanicum	19.15	6,280	HG529494 to HG529928	PRJEB4565	SAMEA2178376
Neurospora crassa	39.23	9,841	AABX00000000	PRJNA13841	SAMN02953583
Rhizoctonia solani AG-I IA	36.93	10,593	AFRT00000000	PRJNA51401	SAMN02981347
Saccharomyces cerevisiae	38.1	5,645	AMQD00000000	PRJNA174690	SAMN02769632
Sclerotinia sclerotiorum	38.45	14,714	AAGT00000000	PRJNA15530	SAMN02953621
Ustilaginoidea virens	38.96	8,426	PGGP00000000	PRJNA414696	SAMN07807409
Ustilago bromivora	20.77	7,408	FMVU00000000	PRJEB7751	SAMEA4497028

Additional File S1. Sanger sequencing results of structural variants (SVs) based on PCR validation for *F. proliferatum* and *F. fujikuroi*

This Additional file 1 contained Sanger sequencing results of structural variants (SVs) based on PCR validation for *F. proliferatum* and *F. fujikuroi*. Results were presented in FASTA format where the read ID corresponded to SV ID in table S3. **A** indicated sequences in *F. proliferatum* strain Fp9. **B** indicated sequences in *F. fujikuroi* strain IMI 58289. The terminal regions in light gray background are primer sequences.

>1-A
AGGGGACAAGAGGTAAGGCGCCGCGCTGACAGCTTCTTGGTCCCTTCAGGGTGACGATGATATCAAAAATCGAGAATAGAGGTTGTCTACGTAGGCTATCCCTGCTTC
GCCTCCCCTGTCAAGCTGGCTACCAGCCCCTGACGGTGCTCCATCACCCAGCGACTTCCCCAAAGGATGGCCCGCTCGGTGCTCCGCTCATCGTCCGAGCCGGCCCG
GTGTTGAGACGCTCTGGTGGTTGTGAGGTGGTTCCTTCTTGCGGGGAAAACTACCCAATTAACAAAATTTGAAGCCGGGGAACTTAATGGGGCCATCTCAGTATGGTAT
CAAGGTTGAAGTAGTAACAAGTGGTTCCATAATTGAAATTGAGCATGCCAAGATCAATCCAAAGGTCTCGCGATCT**CTGCACAGATAGCCTGA**

> 1-B
AGGGGACAAGAGGTAAGGCGCTGCGCTGACAGCTTCTTGGTCCCTTCAGGGTGATGATATCAAAAATCGAGAATAGAGGTTGTCTACGTAGGCTATCCCTGCTTCGCC
TCCCCTGTCAAGCTGGCTACCAGCCCCTGACGGTGCTCCATCGCCCAGCGACTTCCCCAAAGGATGGCCCGCTCGGTGCTCCGCTCATCGTCCGAGCCGGCCCGGTG
TTGAGACGCTCTGGTGGTTGTGAGGTGGTTTCTAAAGGTCGGAGCCACAAAACCCCCTCAATCTGAATGCAATGAGGACCTGTCTTTGTTCCATGAAGCGGTTTGCGTT
TCCTTCTTGCGGGGAAAACTACCCAATTAACACAATTTGAAGCCGGGGAACTTAATGGGGCCATCGCAGTATGGTATAAAGGTTGAAATTGTCAACAAGTGGTTCCATA
GTTGAAATTGAGCATGCCAAGATCAATCCAAAGTCTCACGATCT**CTGCACAGATAGCCTGA**

> 2-A
TAACCTCTCCTATCTCTCAGCCGCGATGCGGAGACACCTCACCCCATTCCCTGATCTGGAAAGACTTCCAATATTGGGCGTAACTGGATCTGGTCTCGCAGATTCAACA
TGTTATACTATATATCTGCCGAGATCAGGCTTCCTTTGGAGTGACAGCATGATCTGGCAGCAGAGTCGACCTGATCAGATATCTAACTCCGTGAAAGACCATGTTGACGA
AAACGGAGAACATTGGTCTGTTGAGGGTTCTCGCCGTGTGTTTTTCCAGGACAGTTTTCTGGCACGACATCCGTGGGAAGTTCAGAAATATCCCCGCCCTAAGCATACA
GCACAATGGAATAAACGGGTTCTCTCGGGCGATACTGTCCGCCTATATGACCCTATGACTAGCCAATATCTTTGCGCAATACCTGGTGAGCGAGTAGTCAGTGAAGCGG
GTTATTTCGGCACGAAAAGTAGCACTGAGCAGGACCTTGATAGAATTCGAATAGGGTAAGCATATACAACTGTTAACAGGATTCGTGCTGACGTACTATCCAAGAACTG
TCTTAAATACTGGCTCTGCGAGCTGTGACTGGAAAGTGTATTATGAT**GTTGTTCAAGGGAGCGT**

> 2-B

TAACCTCTCCTATCTCTCAGCCGCGATGCGGAGACACCTCACCCCATTCCCTGATCTGGAAAGACTTCCAATATTAGGCGTAACTGGATCTGGTCTCGCAGATTCAACA
TGTTATACTATATATCTGCCGAGATCAGGCTTCCTTTGGAGTGACAGCATGATCTGGCAGCAGAGTCGACCTGATCAGGTATCTAACTCCGTGAAAGACCATGTTGACGA
AAACGGAGAACATTGGTCTGTTGAGGGTTCTCTCGGGCGATACTGTCCGCCTATATGACCCTATGGCTAGCCAATATCTTTGCGCAATACCTGGTGAGCGAGTCGTCAGT
GAAGCGGGTTATTTCGGCACGAAAAGTAGCACTGAGCAGGACCTTGACAGAATTCGAATAGGGTAAGCATATACAACTGTTAACAGGATTCGTGCTGACGTACTATCC
AAGAACTGTCTTAAATACTGGCTCTGCGAGCTGTGACTGGAAAGTGTATTATGAT**GTTGTTCAAGGGAGCGT**

> 3-A

ACTCAGTCTGTCCGTTGACGGTAACTGGGGGATCTGTTGCAACCCGAACCTCTTATTCGCATGGGTTACTGAAAAATCCTTGCGGATACGAATATTCCGAGGATCAGGT
GTCTGAAGGCCGTCAGAGTCGTTTGGTGAAGGGCTTCCCTCATCTTGTTTCTGGTAAGTTGTCCTAGGGCTCGAGACCGCTTTGTTCTGTAGTGGCGAAGGCTTTGAGTG
CGGCTTGGGCGAGACTATTGAACGTGAGGTCGGAAGATTGGCTCCATCAACTTTAACGGGGAAACGCCTGGCACGGATTGGCTTTGAAGAGTGCTGGCGCGTTTCGAA
CTGTTGATTTGAACCCTGCGGAATCGGTAGCTCAGACCGGCTGGCAGTAGATGTGGCCTCCTGTTTCAAAAATTGCTTTATCTGAGCCGTGACAGCCATATCAGTGCTTC
CGTTGTCCCGTGCACTGTTGGAAGCATCTTCAAGGTACTGTGCTTCGACAATACGACTCAGCATCAGAGTCAACGGGTTGCCGTCGATCTTGAGGGCGAGGAGTGCAG
TCATCTCGGAGATGCATAATGGCAGATGCTGAATCTTATTCTTTTGGATTCCCAGGACCTTGAGAGACGCCATCTTTGCTATGTCATCTGGGAGGCATTCAATCTTGTTTCT
ACTTAGATCGAGGATCTCGAGACGACTTAACTCCAAGAGCTGCCTTGTAGTTAGCCTTCAGCGGCATGCATATCACGGCTTGAGGGAATATGTACCACAGGAGGAAAA
GTGTGGATGCAATTGCTCCGGATGTTAAGGTAGCGCAAGGAAGTAAATAGAGACAGATGTGGCGGGATGTTTGAAAGATGGTTGTGAGATAAAGCTAGCCTATTGGTG
AGCTAGGTTAGCAGTTGGATATAAGCCTGAATTATGGAAGTACAGACCGCTCAACGTTCGGATTGAGGTTATCGAGAGTATTAGCGGATGAGTCTGAGAGACCACTGTG
GCTAAGGTCGATGGTGACGCCTTGGTCAACAAATAGCCCAGACATGGCCATCACTACTATTCAATACTCCCGTCGACAGTCGCTTCAAGCCTTCTTGTAGCTGCGCCGG
ATCGGGAGATACCACTATGGTGGGTGATGAAGATGTCTGGCCGCTGTAAGTTTAAAGATTAATGCTCTGTTCCTGGTGTCTAAGTGAAGATATTATTCTTTAATGGAGGTG
ACACTAGATCTGGTGGGGCTGTGGTGTTGATGTTGTAAATTGACAGATTCGGTCGTGGCAGGGCGGGGACATTCATTGGAAACGCGGAGTCACATGTTGGCTTAGGTAA
GATGCTTTCGTAGGCCAACATCATAATCATAACTTATTCCGCATTTCCTCATCAATCA**TAGAGGAAAACAGGACC**

> 3-B

ACTCAGTCTGTCCGTTGACGGTAACTGGGGGATCTGTTGCAACCCGAATCTCTTATTCGCATGGGTTACTGAAAAGTCCTTGCGGATACGAATAGTCCGAGTATCAGGT
GTCTGAAGGCCGTCAGAGTTGTTTGGTGAAGGGCTTCCCTCATCTTGTCTCTGGTAAGTTGTCCTAGGGCTCGAGACCGCTTTGTTCTGTAGTGGCGAAGCCTTTGAGTG

CGGCTTGGGCATCACTATTGAACGTGTAAGTTTAAAGATTAATGCTCTGTTCCTGGCGTCTGAGTGAAGATATTATTCTTTAATGGAGTTGACAGTAGATCTGGTGGGGCT
GTGGTGTTGATGTTGTAAATCGACAGATTCGGTCGTGGCAGGGCGAGGACACTCATTGGAAACGCGGAGTCACATGTTGGCTTAGGTAAGGTGCTTTCGTAGGCCAAC
ATCAAACTCATAACTTATTCCGCATTTCCTCATCAATCA**TAGAGGAAAACAGGACC**

> 4-A

CGGATAGTCGTCTTCATGGGAGTAGATTGAACCGATGTTGTTTCCCGTCGATCACTGGGAGGGTTAAATGCCAGTTTTGATGGGCAAGATGCTCTTCTTTGGGGTATGCT
TGGATAGGCAGGGCTTCCAGTCAATTTTGCCAACTTGTCCCATGAGCTTTGAGGAATGAATCTCTTCTTGATTGAAGTTCTGAGAAGATACTCAGCATGAGAAGGTGTTG
TGGGCGTCGTATCAGCCTCGCGACGACGTCCTACCAAGGTTTGTGGCGTCCTATTATTGAGTGTTGTTGGGGATACGACTTCAGTGGCTTTCGCTTTGCCAACCTCTTCCT
CTTCGTCGCTAGATTCATCCACGGAGGCAGTGACACAGCCTCCGTGGTTTTCAGTTACAGCTAGGCTGCTGGTGACATCGGCTGTCGCAGTCACACCCATGTCCTCATCT
TCTGACCCTGTGGCTGATTGACATTCGCTGACCGAATCAGATGGCGTGGTGTCAATCATCGAAGAAGGTCGGCAGTCAGAAATCTCGAATCCACGAAACTCCTTGCTTG
AGATAGATGACAGAGGACTTGTCACAGCGATTTGGTCAATTTCCATTGCTGATTCAGACTCTCGTAGGCTGCGCCCCTCCTCTACTGGTGTGCTGGCCATATCATCGTTG
GGCGACGACAGGCCAGACCAAGTGTCCTCGTCTCTGGATATGGACACATCTTGGTCTGATGCAGTCTCAACAGCTTGATTGAGCGTTGGCCGCATTTTAAAACAGAAA
CTGTCATCTTCTTGGGTCTCAAAAGCGGAGCGAGGATTCCTGACAACTTCATCTT**GGGCTCTCAAATCAGTT**

> 4-B

CGGATAGTCGTCTTCATGGGAGTAGATTGAACCGATGTTGTTTTCCGTCGATCACTGGGAGAGTTAAATGCCAGTTTCGATGGGCAAGATGCTCTTCTTTGGGGTATGCT
TTGATAGGCAGGGCTTCCAGTCAATTTTGCCAACTTTTCCCATGAGCTTTGAGGAATGAATCTCTTCTTGATTGAAGTTCTGAGAAGATACTCAGCATGAGAAGGTGTTG
TGGATTTTGATGGCGTCGTATCAGCCTCGCGACGACGTCCTGCTAAGGTTTGTGGCGTCCTATTATTGAGTGTTGTTGGGGATACGACTTCAGTGGCTTTCGCTTTGCTGG
TGACATCCGTTGTCCCAACCATATCTTCATCTTCTGACTCTGTGGCCGATTGGCATTCGCTGGGCATTTCTTCAGTGACGAGCACTATTGCGTCTTCCGCTGACACTTGAC
TAGGACAACTTGTGAGTTCTGAAGCAGATGGCGTGGTGTCAATCATCGAAGAAGCACTGGCGCTATCTGGTCGGATGTGATCGGAGGCAGTGACAATGCTCTCGTGAT
TTTCAGTTACAGCCAGGCTGCTGGCGACATCGGCTGTCACAGTCACACCCATGTCCTCATCTTCTGACCCTGTGGCTGATTGACATTCGCTGATCGAATCAGATGGCGTG
GTGTCAATCATCGAAGAAGGTCGGCTGTCAGAAATCTCGAATCCACGAAACTCCTTGCTTGAGATAGATGACAGAGGACTTGTCACAACGATTTGATCAATTTCCATTG
CTGAGTCTCGTAGGCTGCGCCCCTCCTCTACTGGTGTGCTGGCCATATCATCGTTGGGCGATGACAGGCCAGACCAAGTGTCCTCGTCTCTAGATATGGACATATCTTGG
TCTGATGCAGTCTTAACAGCTTGATTGAGCTTTGGCCGCATTTTAAAACAGAAACTGTCATCTTCTTGGGTCTCAAAAGCTGGGCGAGAATTCCTGAAAACTTCATCTT**G**
GGCTCTCAAATCAGTT

> 5-A

ATGACAACCATGAATCAGGAGTTAGTGAGCCTTGTGCCGCCCAGTACTGGTTGAGCTCCATAGCTTCATAGCTCCACTAGCCAAGCAGTCAGATAGTAGCCAGATACC
GCTGAACCATGCCAGACCGCACAGACGATGTGTTTCAGTTCTGAGACTACACCCTGATGTGTGCAATCGCCTGCAAACATGTCACTTTCACATAAGTAACTCGATTTGA
TCGATACGTACGAACTTTGGTTCGGCTGCATATTATTGCAAGGTGCTATGCCCGGGGAATCTGGGAATAGATCTGACTTATTATAGTGGTATTTTGTACATTAGAATCAACT
CAACGTGTCTGAGATTGAGGTTGAGATTCGGCGAATTTTCTGGGCCTCATCTACGAATACCTGACCGGCCTTACTATAACAAGGCACGCCGATCTAGGTGCGGGGGTAG
CACATGGCATCGCGGATAACACAAGGCACAGATGACCGAATATCGACCGGCTTGTTTGTGCATAACATCTTCCGGAAGTACCAAGCTTTTCTCAGCAGACCAGCGCCG
AGAAAAGCTTGGTCCAGACGGGCATATCGGTCAGCAAAGCGCCGGGAAATGGCTCACTCAGTCGTTGGTCACTGAACATATAGTGCGCTTACCGAACCGACCAAGCC
TTGAGGGCATATGCTCTATAGTCATGCTCATGCCTGGCATAGCTGAGAGCATTTGGCG**CACAGGGTCTTCTTGTT**

> 5-B

ATGACAACCATGAATCAGGAGTTAGTGAGCCTTGTGCCGTCCAGTACTGGTTGAGCTCCATAGCTTCATAGCTCCACTAGCCAAGCAGTCAGATAGTAGCCAGATACC
GCTGAACCATGCCAGACCGCACAGACGATGTGTTTCAGTTCTGAGACTAAACCCTGATGTGTGCAATCGCCTGCAAACATGTCACTTTCACATAAGTAACTCGATTTGA
TCGATACGTACGAACTTTGGTTCGGCTGCATATTATTGCAAGGTGCTATGCCCGGGGAATCTGGGAATAGATCTGGCTTATTATAGTGGTATTTTGTACATTACGGAGGTC
CCTGCCAAAAGTTTGGTCTCGACCCCCTGCTCATAAAATAGAGGATTAGGCGATTACAGCTAATAAGGTACTTCAGTACTTTAGTCTTATCTTATTAGGATTAGAAGCTCT
ATAACCTTTCCCCTAAAGTCTACTATAATTATAATTATAGCGCTTACTTTACTATCAATAAGAGACCTCTTGTACCTTCTGGGTATCGATAGGGGCATTCCCTGCTAGCTTCA
TTTGGCTAATAGGGGGTCAGGATAAAACTTTTGGCAGGGACCTCCGTAGAATCAACTCAACGTGTTTGAGATTGAGGTTGAGATTCGGCGAATTTTCTGGGCCTCATCTA
CGAATACCTGACCGGCCTTACTACAACAAGGCACGCCGATCTAGGTGCGGGGGTAGCACATGGCATCGCGGATAACACAAGGCACAGATGACCGAATATCGACCGGC
TTGTTTGTGCATAACATCTTCCGGAAGTACCAAGCTTTTCTCAGCAGACCAGCGCCGAGGAAAGCTTGGTCCAGACGGGCATATCGGTCAGCAAAGCGCCGGGAAATG
GCTCACTCAGTCGTTGGTCACTGAACATATAGTGCGCTTACTGAACCAACCAAGCTTTGAGGGCATATGCTCTATAGTCATGCTCATGCCTGGCATAGCTGAGAGCATTT
GGCG**CACAGGGTCTTCTTGTT**

> 6-A

ATTTATGTGATGCCGCCGGCATCTGCATGGCGCAGGCGTGGGACCAGTTCAAATACCGCGGCGACGGCGGTAAATCATCAACCTGCTGGTGCTGATCAACTTCCGAGC
AAGAGAATGGGTGCTCATCAACACTTTTATCGACATTGTAGCCATATAATGCTTGCAAATAGAGAGCAATTGCCCTGCAGTCACAGGGTATCACATTGAAGGGGAAAGG
AAGCAGGGGTTGTGTTAAAATCCATCATCTGCCTTAAGCAATACGAAGAGGAGTGAAGGCTTACAAAACTCCCAACCTGCGGCAACCTCGCTCTTAGCGCGGCAACG
CACTGTCTGTTCCCTGTCATCCTAAATGGATCTCTGCTTTGGCGTGACATGGCAGCTGGGTTGCTGCTGATCGTTTGATCCCGATGAGATGAGCAATCGGCCGAGTCGAG

CTCACAGACATTTGCCCCTGACACTTGTGGGGAGCGGAAAACATCAAGCAGAGCAGAGCCTGAGAAGATCTAGGTAGAGCTCACGCCCTCTACCCTTGTAAACGAAC
TTGTGCTAGGTGTGGCGAGTCATGAACTCACACCATTTCTTCAAGTTCATAATGTTGATATCCCCACTAAAGCTTCGTTTCTTTGACGATGTGTATACTTGTTCGAGATCTA
GGGTGGTTTCAGTGGTTCGCAGCTCCTCAGACCGGAACGTCCAGTGTTCCGTTGTCGATCTGCCAAGTGCCTTGAGTTCCTCGACTTCATAATCACATGTTCAAAACAA
GGACGAACGGAAACAATTAAGTTTGCCAACGGCATAACAGTAGTGTGAACAGTAAACTCAGTTGAAGATTGCTTCTGCCGTTCGCTACTGTGGTTGGGTTAAGGCTGG
CAGACATCCTGCAGGGGACTCAAACATGTACAGATACAGAGAATGATTCAGGTTCAGGATGTCATTTCCCTGGCACGTTGGCGGGTACTGTACCAGGGAATATTCAGTC
ACGTCATTAAATTACTTGTCGAACTACCCTAAAGAACAATTTCTATCCTGCCGTGAATCAGTATTCAGCAAGTCCCTCATGACCAACCGGCATCGATGAGTTGGCACGGA
TGTGGCCCATGACGGACGGAAAGAGACGCAGTTGTTATAAAGAAGCAATGCAGCGGCACATGGAAAAGCAAGTTTTAACAAGTTCCTCAGCATAACTAGTCGCCGAG
TCCCTTGCAGTGAGGCATATCAAAATGAGGGGGTTTTGGGCGGCACAACTATCATCAGAACTGGGGTATTCAACTACCAATTTGCACGACGATCAATTGATCGAGAATC
TTGCGCCAAGGCTCGACCTGCTCACTGCTTTTTGTATATTGTTGCCTGAACAGGCCTTGCTCCCAGGTGTCATTTCGAATCCCCACACAATACTGATATAAGCAGGGTTG
CGGATTTGCAGCCCCAGCCCCAGCCCAGCCTCGCTCAGTTCAACTCGGCCTATCGCTTGACTCTTGATGAATGACTGGGCTTCGAACTTGGCAGAATGGAGTATGCTCA
CAGTCTTTCAACGACATGACTCGATAAAAACAATTGCGTGCCGTTCCACGAGTCCACAGTTGTTGCGCCTGGCCATTATTGTTCTTCTTACGGACTCACTCCTTGTTCCC
GCCGAAATGTGATATATGACAGAACGCTAGCTGTCTAGGGACCCCCGGATCTCAGCAACATATCACAAAACATCACTCCGACATGGGTAGCTTAATACGAGAATCTCA
GAACGTACAGTATGTGAACAGAAACAATGGGCGACAGGGGAAAAGCCAACGTCTATCTTGTCTGGTTCGATGGATTAACACTTAACGGGGGTTTTAGGCGTCTTTTCCC
GCCCTTTACTACACGCCCGCCATCCTCTCAGGGTTGCAGGAATCCCCTCAACGTGCATAAAATCCTGGACGCGGATCTTCTCAGTTGGAGAAGCCCATACTCTGCGTTA
CATCGTAGGGGGGCTAAGCGGCTCGGAGTTAGCTTTTGCCATGGTAGCTATACCGCTGCCCACCATGATGCCCTGTTCTATCTGCTCTACGACGTCTCGTTCGCCTCACCA
TCTTCACATCCCCTGAATAACAGTCGGTATATGGTCAACCATATTGGTGCCTGGATGCCTCTATATTGACTATATTGAGCGTAAATTATTCTCTGCGAACTAGGGATTTCCT
TGTCTATCAGTTCTCCTTGGTTTACATATACCCAGGCCTATGCGGAACCGCAATATTAAATTCGCTAAAACAAATTGTTGGAGCTGCCTTCGTCCTGACTAGGAAATGGA
AACGAATCGGTGAGCTCTCCCTTGATTCCTCATGATCCCTCACTGCCTTATCCTTCGTGCGCTTCCCAAGGGTGTTGGTTGATCAGGTGTGAGAGACTTGTGTCTTCTCAA
CTGACGCGTGGTGACCTGAA**TCAAGCATGAGTTGTCGAT**

> 6-B

ATTTATGTGATGCCGCCGGCATCTGCATGGCGCAGGCGTGGGACCAGTTCAAATACCGCGGCGACGGCGGTAAATCATCAACCTGCTGGTGCTGATCAACTTCCGAGC
AAGAGAATGGGTGCTCATCAACACTTTTATCGACATTGTAGCCATATAATGCTTGCAAATAGAGAGCAATTGCCCTGCAGTCACAGGGTATCACATTGAAAGGGAAAGG
AAGCAGGGGTTGTGTTAAAATCCATCATCTGCCTCAAGCAATACGAAGAGGAGTGAAGGCTTACAAAACTTCCAACCTGCGGCAACCTCGCTCTTAGCGCGGCAACG
CACTGTCTGTTCCCTGTCATCCTAAATGGATCTCTGCTTTGGCGTGACATGGCAGCTGGGTTGCTGCTGATCGTTTGATCCCGATGAGATGAGCAATCGGCCGAGTCGAG
CTCACAGACATTTGCCCCTGACACTTGTGGGGAGCCCATACTCTGCGTTACATCGTAGGGGGGCTAAGCGGCTCGGAGTTAGCCTTTGCCATGGTAGCTATACCGCTGC

CTACCATGACGCCCTGTTCTATCTGCTCTACGACGTCTCGTTCGCCTCGCCATCTTCACATCCCCTGAATAACAGTCGGTATATGGTCAACCATATTGGTGCCTGGATGCC
TCTGTATTGACTATATTGAGCGTAAATTATTATCTGCGAACTAGGGATTTCCTTGTCTATCAGGTCTCCTTGGTTTACATATACCCAGTCCTATGCGGAACCGCAATATTAAA
TTCGCTAAATCAAATTGTTGGAGCTGCCTTCGTCCTGACTAGGAAATGGAAACGAATCGGTGAGCTCTCCCTTGATTCCTCATGATCCCTCACTGCCTTATCCTTCGTGCG
CTTCCCAAGGGTGTTGGTTAATCAGGTGTGAGAGACTTGTGTCTTCTCAACTGACCGCGTGGTTACCTGAA**TCAAGCATGAGTTGTCGAT**

> 7-A
GCCTCGGGATATGGAGGTCTCTATGCCGAGCGGGGCTTCGTTTCGTGGGTGAGGCCTTGGCCAACTCTTGGTGAGACTATCGTCAATGTCGTCACTTTTGCCAACTCGT
GCAATGCCTACATTCCGATACAGGAGAAAGAGGCAGCTTGTCGATTGTCTAGTCACGAGATCCGATTCAATGCTTTTCTTGACGACTATTCTTTGAATCAACTAAACCCA
TGCGCCTCAAGCCAACATTGGTCCAGAATATGCCAACATACCTATCGATCGATGTCTTTAGACCAACAACTCCTCAATTTTGCCAAGCCTTACAATTGTCCCCTGCTTTAC
CCAGTGACATACAGAATCGAGACCCTTGCAGGGATATTCTGACGGGCAGTTATCTCAACACCAACTAGAGTCAAAAAGTCCTCCTATACCTTCCCGATTGGGATGTCCA
GCTATTGACACGATGAAGGACTTTAGGCGCGGTCTGAGGTGACTCTCGAAAGAGCGATCGATTGGTAGTACGAGCTATGATATTCTGTAATCAAGATAACAAGACGGTT
ATGTGAGGATATCCCTGGGATCTAGTTAAAGACCTCGGATTATTATTGGTCACGGTTCCTGACCAAAGGCTTGGTGGTCGAATCAGATATCTCTCGCAGATGTGGCCATG
GTGCAATTGAAAGTCTGAAACTAGATTCTAGATTCAGTGCTAATCAATACCAATACCAGAACCTAATGATTTTCTCATCTGTCCGACACCGAGGATTTCAATAACCCGTC
GGCCTCCAAATCTCTAAAGCTCCGACACCAGCAACGCCACAACGCACAAGCCACCCG**CCTCATCCCTGTTCTCGA**

> 7-B
GCCTCGGGATATGGAGGTCTCTATGCCGAGCGGGGCTTCGTTTCGTGGGTGAGGCCTTGGCCAACTCTTGGTGAGACTATCGTCAATGTCGTCACTTTTGCCAACTCGT
GCAATGCCTACATTCCGATACAGGAGAAAGAGGCAGCTCGTCGATCGTCTAGTCACGAGATCCGATTCAATGCTGTTCTTGACGACTATTCTTTGAATCAACTAAACCC
ATACGCCTCAAGCCAACATTGGTCCAGAATATGCCAACATACCTATCGATCGATGTCTTTAGACCAACGACTCCTCAATTTTGCCAAGCCTTACAATTGTCCCCTGCTTTA
TCCAGTGACATACAAAATCGAAACCCTTGCAGGGATATTCTGACGGGCAGTCTAGGGGAGGAAAGAAAACTCTGGACCTTGATCCTAGATGGCAAAAAGACTTAGGT
AAATTTGCATAAATTTAGGATATCTTTTGCTGGGTTTATTTTGACCCCCTATATCAAGGTCCAGTGTTTTCTTTCTCCCTGAGGGCAGTTATCTCAACACCAACTACTAAGA
GTCAAAAAGTCTTCCTATACCTTCCCGATTGGGAAGTCCAAACATTGACACGATGAAGGACTTTAGGCGAGGTCTGAGGTGACTCTCGAAAGAGCGATTGATTGGTAGT
ACAAGCTATGATATTCTGTAATCAAGATAACAAGACGATTATGTGAGGATACCTATCCATGGGATCTACTTAAAGACCTCGGATTATTGGTCACGGTTCCTGACCAAAGG
CTTGATGGTCGAATCAGATATCTCTCGCAGATGTGGCCATGGTACAATTGAAAGTCTGAAACGAGAATCTATATTCAGTGCTAATCAATACCAATACCAAAACCTAATGA
TCTTCTCATCTGTCCGACACCGAGGAATTCAATAACTCGTCGGCCTCCAAATCTCTAAAGCTCCGAAACCAGCAACGCCACAGCGCACAAGCCACCCG**CCTCATCCCT
GTTCTCGA**

> 8-A

GTCTCCTCTTTCCTCACTTGAATCTTGCCGTCCCGTACCGTGCTGCTCATCTCCCTCTCATACTGTGACGAGCACTGACACCGCCAGAACAATATCCATTAAACACTGGC
CCAGAGGAGGACCTAAAAGACCGCCAACCATCAACTAACTCTATATCTCTGGCTGGTTTCAAGTGTAACTCTTCCGCAAAGGCAGGCGACTTGCGTTGAAAATGTTTGT
TTGTGCAATGCATATGCACATATATGCACATAGGCTGATGCAACTGATGCAACAGCAACACACTAACGTGGGTTATGCCAGGGATACCCACGACTAATCTCCAGCCACG
CATTCTCTGTCCTTGACTGTCAGTGGCCAACTGTACGATGTGTATGCCCAACTCGACAATCCAAATGCTTGATCCAGCATCTCTGGAATACGACCTGATGCATCCATGTTT
CAAGATTGGCTGCCAGTTGTAGGCAGCCTAGGGGAGGAAAGAAAACACAGGACCTTGGTCCTAAATGACAAAAAGACTTGGGGTAATATAGCCTAAGTTTAGGATGA
CTTTTGCTAAGTTTATTTTGACACCCTACAGTAAGGTTCGCTGTTTTCTTGCCCCCGGAGGGCAGCCCTTCAAATGCTGTGCACCACACCACTTTCTCTTGTGCCGAGCAT
CAACTGTCAGCGGCAATGGAATGCATACGTACGTACTCCAAATCCACGTTGTTAACCGCCAGCTAGGACTGGTTGTTTCAGTCGATTATTCCTCGGCTCACACAACCCAT
CCATGTTCAGTTATGGATACTTGATCGTTTAATACAACCTTGAGGGTCGCATTGCCCGGACATGGGCTCCGGCAGAGTCATGTTGCCTACCATCCTTGACATTTCACAAGT
CATGTCAACTCATCTCGGTACTTTATTCGAAACTGCCTAGCTGTCAAAAACATCTGACATTCGCAGGTAGCCACAATGCGCTGACCAACTGCATCACGGCATTCGCTCC
ATCACGAAGTCTATCAGAACAAAGAAACCCAACTCCCTGGCCACGGCGCTGCGGGTACCATTTTGCTGCATTCCGGTCTCCTCCAGAAATTCACCGTGCGTAA**AAAGG
TTCTGTTGGTTC**

> 8-B

GTCTCCTCTTTCCTCACTTGAATCTTGCCGTCCCGTACCGTGCTGCTCATCTCCCTCTCATACTGTGACGAGCACTGACACCGCCAGAACAATATCCATTAAACACTGGC
CCAGAGGAGGACCTAAAAGACCGCCAACCATCAACTAACTCTATATCTCTGGCTGGTTTCAAGTATAACTCTTCCGCAAAGGCAGGCGACTTGCGTTGAAAATGTTTGT
TTGTGCAATGCATATGCACATATGCACATAGGCTGATGCAACTGATGCAACAGCAACACACTAACGTGGGTTATGCCAGGGATACCCACGACTAATCTCCAGCCACGCA
TTCTCTGTCCTTGACTGTCAGTGGCCAACTGTACGATGTGCATGCCCAACTCGACAATCCAAATGCTTGGTCCAGCATATCTGGAATACGACCTGATGCATCTATGTTTCA
AGATTGGCTGCCAGTTGTAGGCAGCCCTTCAAATGCTGTGCACCACACCACTTTCTCTTGTGCCGAGCATCAACTGTCAGCGGCAATGGAATACATACGTACGTACTCC
AAATCCACGTTGTTAACCGCCAGCTAAGACTGGTTGTTTCAGTCGATTATTCCTCGGCTCACACAACCCATCCATGTTCAGTTATGGATACTTGATCGTTTAATACAACCT
CGAGGGTCGCATTGCCCGGACATGGGCTCCGGCAGAGTCATGTTGCCTACCATCCTTGACATTTCACAAGTCATGTCAACTCATCTCGGTACTTTATTCGAAACTGCCTA
GCTGTCAAAAACATCAGACGTTCGCAGGTAGCCACAATGCGCTGACCAACTGCATCACGGCATTCGCTCCATCACGAAGTCTATCAGAACAAAGAAACCCAACTCCC
TGGCCACGGCGCTGCGGGTACCATTTTGCTGCATTCCGGTCTCCTCCAGAAATTCACCGTGCGTAA**AAAGGTTCTGTTGGTTC**

> 9-A

AGGTCGGTGTCGGTATCGTCGCCTCTGGTGTCGCCAAGGCCAAGGCCGATCACATCCTGATCTCTGGTCACGATGGTGGTACTGGTGCCTCTCGATGGACTGGTATCA
AGTATGCCGGTCTTCCTTGGGAGTTGGGTCTGGCTGAGACTCATCAGACTCTGGTTCTCAACGATCTTCGTGGTCGTGTCGTTGTCCAGACTGATGGTCAGCTCAAGACC
GGTCGTGATGTTGCACTGGCTTGTCTGCTAGGTGCTGAGGAATGGGGCTTTGCCACCGCTCCTCTCATCGCTATGGGCTGTGTCTTCATGAGAAAGTGCCACTTGAACAC
TTGCCCTGTTGGTATCGCTACGCAGGATCCTGAGCTTCGCAAGAAGTTCACTGGAACTCCTGAGCACGTCATCAACTTCTTCTACTATGTTGCCAACGAGCTCCGGGCTA
TCATGGCCCAGCTTGGTTTCCGAACCATCAACGAGATGGTTGGCCACGTCGAAGTCCTCAAGATGCGTGATGACCTTCGAACCCACAAGACAGCCAACATTGACCTGT
CTCTCCTCCT**GACACCCGCACACAAGC**

> 9-B

AGGTCGGTGTCGGTATCGTCGCCTCTGGTGTCGCCAAGGCCAAGGCCGATCACATCCTGATCTCTGGTCACGATGGTGGTACTGGTGCCTCTCGATGGACTGGTATCA
AGTACGCCGGTCTTCCTTGGGAGTTGGGTCTGGCTGAGACTCATCAGACTCTGGTTCTCAACGATCTTCGTGGCCGTGTCGTTGTCCAGACTGATGGTCAGCTCAAGAC
CGGTCGTGACGTTGCACTGGCTTGTCTGTTAGGTGCTGAGGAATGGGGCTTTGCCACCGCTCCTCTCATCGCTATGGGCTGTGTCTTTATGAGAAAGTGCCTCGAAATTA
GCACTATGGTTAGGACATCCCAAGGACCTAAGCCTATCTCTGCTATCAACATTGGGGACACACTATATGATCCCAATGATAACCCTGTCCTATGTCTTGCCGTGGCTCCTG
TTCAACAAGGTCGTTTGAAGGAGATACGCTATAAAGAGTTCGATTCACGACGCAACGTGTCTTTCAAGTGTACGCCTGATCACATATTGCCCCTTCGAACCTATGGCAC
CAGGCCTTCGGTCAGCAAGATCAGTGTGACTTGGTTCACGTATTGTGATAGACACTCCCTTGATCGCCAGGCGGACAATCTTCGCTTTGAAGATTTGGTTGACACTATCT
ACAGAGACTTGATGGATAGTCATGAAGTTGAGATTCCTGGACAAGCCGCGGCCCATGGGTATATCGATACTATTCTAACACGACACTACCACCAAGGACAAGACGATC
GTTACTCGAACAAGATCGTTGAAGTTCTTTCTCGATTGGCCAACCATGAACTTGAAAACAATCCAGACCTGGTTCGTGAGGCTATGCACGATGCTGTAGATCTGTACTTT
GAAAATCCTCCCAATCTCGTGGACGATGCAGAGTACGATGATGATGCCCCTACCATCGATCTTGGCCCTGACTTTGTGGCTCTTCGAGACGCTACCCTGGCAAGTACAA
CTGCTGGCAGTACTTATCGCCGCACACCTTCGTTGAATCAGCCAGACTCACAGTCATCTCGCCAATCTCAGCAAACCCAGCGGGACTCTCAACAGTCTGGAGGCATAC
CTCAAGGCTCTGGCGGTATATCTAGACAAGTATCGTTGAATACCCAAGCATCCAGCTCCTCGTTCGTGGATTCTTCGGCCATAGCTCTATCTGAAGAGGCCATTCAACGA
TTCAATCGGGTAAAGACATCTATCAACCGTAAGCCTTGTCCATGTGGTGGTATTCGCAAGATCTATCGCCAGTTTGCCTCTGAAGAACAAGCCCAGCTTGTTCAGTCTCT
ACTACTCAGTCAACATGCTCATATGGTAGACCCATATGTTGTTCGAGATGGAGATGAGATCAATGTTTCTGTCGAGACATTTGAGGGCTTCTGCAATAAAGAAGCTAAAC
GGATGCACCTCAAGCTGCATCGTGCTCCATTGACCTTTGTCCCGGCTCCTAGTGGACAAGATCAAGCAACAGATGAAGCTCTACCAATCGACCCATACTTCCTTGGTTTA
TGGCTTGGTGATGGTGGAGCTGAGGGTCCTTTTATCTATGGCAGTGACCATGAGACTCGTGTTTGGCTATCCAGCTATGTTGATCGTTTGAATAATGGCAGACCAGCTGGT
ACTAGGCCATTGAAACTTAGAGAGTATCGAACGCAGCAAGCTAGTGACTCTCTGCGTAACTCTAACTATAGGGCTAGAGTTGATGTCTTCCGTTGGGCCATTACTTCAG
AGTTACATGCACCTGGCCGGCCATGGAGTCCCATTCGAGAAGGCTTACAAAACCTTGGGCTTCTCAACAACAAGAGCGCTGGTATCCCAGACTGCTACCTCCAGGCCA
GCAAGCAAGCACGCCTGGCAGTCCTGGCAGGTCTTATAGACGCTGATGGTTGCTATGTCAAGTCTCATAATACCTACCGGTTTATCCAGCACGGTGCAGGACACAGGA

AGATCGTCGAAGATGCTAAGACTTTGGCTCTTAGCTGTGGCATTTCAGTGACAGGTGTTGACACGTCGACACCTCTCAACCAGTGGAACAACAGGCATGGGCCAAGCG
TCCAGTACGTGATCTATCTAGGTAAAGGTTCAAACGAGTTCCAGAACCACCTGTTGATCCCACGCAAGAGAATGAACCCAGCAAAGACATTCTATACGCACGATGCAC
GACCTTTTACTGTCTCGGACGTCGAGGATGGTCAGTACAGAGGAATCCAGGTTTCTGGAGGGCTATTCCAGCTCCAGAACCGCCTCGTTACTCACAATTGCCACCTGAA
CACTTGCCCTGTTGGTATCGCTACCCAGGATCCTGAGCTTCGCAAGAAGTTCACCGGAACTCCCGAGCACGTCATCAATTTCTTCTACTATGTTGCCAACGAGCTCCGG
GCTATCATGGCCCAGCTTGGTTTCCGAACCATCAACGAGATGGTTGGCCATGTCGAAGTCCTCAAGATGCGTGATGACCTTCGAACCCACAAGACAGCCAACATTGAC
CTGTCTCTCCTCCT**GACACCCGCACACAAGC**

> 10-A

GCTGCCCATCATAAGTGGAAGGTTATCACGATTTCTGCCAATTGCTAATGCATAGTAGGTCATAGGGGAGCTTCCATGAGAATACTATATCCTGTAGGAAGCGCAATGG
CTTATTGCAATGTGTGCGAGTTCTGATTGCATGAACATGGATGTTTTCTCTGCCCTTGTCCTTTCATTCATTCAATTATAAGGATCATATTGGCTGTCATGACAAGACAGTT
CATCATGTTGGCGGGGTTGAGGGGTTATCTTTCAACTTTAGGGTAGCTAAATTGCACGGCTCACACCTCGCTGGGCGGCACTTTTGTCATGAGGATGATTCTACAAGGTG
GAGAAGAGCCTTGTTACTAGGGATGGAGAGTCCCTGTCGTTGCCCACACGGAGATTCTGGTCCGGCGATTCATTGTGCGCTGATCTCCTTTCTGTATATCCTAAAGCACT
ACACGGAAGATATCGAGATGAAGCAAAATTGCCATTCGTTGTCGGCAAGGTGAGGCCATCGTGAAGTTCGGGGAAGATGTCACAAACGGACATTTTTAAGGATGAGC
CGCATTTATGGAACCCCGGCCCCGGTG**ACCGAGATTGGGGTGA**

> 10-B

GCTGCCCATCATAAGTGGATGGTTATCACGATTTCTGCCAATTACTAATGCATGGTAGGTCATAGGCGAGCTTCCATGGGAATACTACATCCTGTAGGAAGCGCAATGG
CTTATTGCAATGTGTGCGAGTTCTGATTGCATTAGCATGGATGTTTTCTCTGCCCTTGTCCTTTCATTCATTCATTTATAAGGATCATATTGGCTGTCATGACAAGACAGTTC
ATCATGTTGGCGGGGTTGAGGGGTTATCTATCAGCTTTAGGGTAGCTAAATTGCACGGCTCACACCTCGGTGGGCGGCACTTTTGTCATGAGGGAGGATCTGATATCAAA
GATCCGTAATTACATTTCAATCTAGCTCCAGCCACAAAAAATCATCAAACCCCATTAACTGCATCGTGTCTTATGACTTGAATGTGATGGAGTATGAGGTCTGGGCGCCC
CGATTGAGGACTATTGGATCATGAGAAGATATATCGAAAAGCGTTGCAGATAACGGCTGGTCTAACACTCCACCATTGGCAGCCTTGTCAAGCTGATGTGATGATAGCA
GTAGAGCGAGGGTATGTATTTTCATCTACAACTCCAGACTGAACAAATAGCCAGCGATCGTTAAGAAAGAAGGAAATAAAGCGACATGAATTGCTGGCAGCTACCAGC
CATACAGACTGGCACTGCATAATATGGTAGCTACCTAAGTAGGTAGTGATACTATTCAGAGGTACATACCCAAATGACGTCCTGCCGGGCCTGGAAATGACGGTGTCCT
GTTCATCCACGCTTGCCTTTGTTTGGACATAAAGCCAGGCGTACAAAGGACACATGACGGATCTATACAGTTGACTGATGTATGTCACGAAAAATATGTTGTCCAGGGC
ATGATCGACTTGGCATGATGATTCTACAAGGTGGAGAAGAGCCTTGTTACTAGGGATGGAGAGTCCCTGTCGTTGCCCACAAGGAGATTCTGGTCCGGCGAGTCATTGT
GCGCTGATCTCCTTTCTGTATATCCTAAAGCACTACACGGAAGATATCGAAATGAAGCAAAATTACCATTCGTTGTCGGCGAGGTGAGGCCATCGTGAAGTTCGGGGAA

GATGTCACAAACGGACATTTTCAAGGATGAGCCGCAATTATGGAACCCCGGCCCCGGTG**ACCGAGATTGGGGTGA**

> 11-A
CTCTAATAGTGATGCGAGGTGGAGGAGGTCACACGGCTGGCCAACTGACACATGCAGATCTACGTAGCCTGTCTCGCAACTGCTTGGCTCTATCACCAGACATGTACC
AACTTTGTTGCCTCACTTACTGAAGGTAAGTAACTTACCCTGCGGCCTTGTTGTATTGTAGGACTTGCTGGTTTCACGATTAATAAGCTACAACGGGACTTGGAAACGAC
CAAACACTTTGGCCGCTATGACCGCCTTTTGAGCTGATGTTTCTTGGTCTTATTGGCGTGCTCTCTATAAACACATCATCTTGAAGCGGCTTGTGCTTTATGTACCACCTAG
TAAAGAACTTACGTTTCACGATCAACTAACTAATGTAGTTGCAGTATCTAACTGCGGCTCCCAACAGATGCTACTAGCTGCATCGGGGTTGTCTTAGATCCAACACAAAC
ACCATCCTCAGGTTCAGCTTACTAGTAAATTCGTAGTGCGATTTCATTGACTATCTTCTGTACGCTGACCATCTAGCCCAGCAACCCAGCAACCCAGCATCTCTAGCCTC
GTCAATTTAGTCATTCAGTCGTACGAGCAATCTCCGCAAACAAGCGGCTCCTCCCTTCCCCCAAGTTCCCTACCTATAATTAACGGCATACGAATGAATGAACACCATCA
TTGCTTCTTCCCACAGACATTATTGCGCCCGC**GACCCAAACAAAACGCT**

> 11-B
CTCTAATAGTGATGCGAGGTGGAGGAGGTCACACGGCTGGCCAACGGGCACATGCAGATCTACGTAGCCTGTCTCGCAACTGCTTGGCTCTATCACCAGACATGTAC
CAACTTTGTTGCCTCACTTACTAGGTAACTTACCCTGCGGCCTTGTTGTATTGTAGGACTTGCTGGTTTCACGATTAATAAGCTACAACGGGACTTGGAAACGACCAAAC
ACTTTGGCCGCTATGACCGCCTTTTGAGCTGATGTTTCTTGGTCTTATTGGCGTGCTCTCTATAAACACATCATCTTGAAGCGGCTTGTGCTTTGTGTACCACCTAGTACGG
AGGTAGCCGCGAAAAGTTCCTGCCCAAGGTGACTTACGCCACAGTAGCTATTTTTACTAGGTACAGAGAGATACAGAGAGATAAGATTACTTATAGAGGGATCCCTCTA
TATCTCTCCTATAGTCTTAGGGATTATAGAGGCAGTAAGCCTGTATAGCTATTGCTTATTTCCCTATAGCGAGATGATGATCAGTGCCTCTTATACTAGCGAAAGTATCCCTT
GTATCTGTGGCTGGTAAGCTTCTTCAGCCACAGTTTTCTCGGGCAAGAACTTTTCGCGGCTACCTCCGTACAGAACTCACGTTTCACGATCAACTAACTAATGTAGTTGC
AGTATCTAACTGCGGCTCCCAACAGATGCTACTAGCTGCATCGGGGTTGTCTTAGATCCAACACAAACACCATCCTCAGGTTCAGCTTACTAGTAAATTCGTAGTGCGAT
TTCATTGACTATCTTCTGTGCGCTGACCATCTAGCCCAGCAATCCAGCAACCCAGCATCTCTAGCCTCGTCAATTTAGTCATTCAGTCGTACGAGCAATCTCCGCAAACA
AGCGGCTCCTCCCTTCCCCCAAGTTCCCTAACTATAATTAACGGCATACGAATGAATGAACACCATGATTGCTTCCTCCCACGGACATTATTGCGCCCGC**GACCCAAAC
AAAACGCT**

> 12-A
TGCCCAGTGATAACGACTAAGTAACGGTACTTGTGATGTGGGTGGGGAGACAGTGCGGGCTCGTGGCGCATACTAGTAGATAGCTACTTATGAAACAGCGATATGCCA
GGCATGTTCTCCATTTGGTGTAACAGTACTAATGCTGGAACGATGACGCTCTTCTTGTTGCACACTGTATATCTGTGGTCGCGAGAGCGGTGAAGCTCCTTTCTATAAGTA

TCGGTACATCAGCCCCCGCGTTACCTACATCTGAGCACGCAGCACGGAACACTACTAACTTTACGGTCATGAGTACAAGGCGGGGTTTAGATCACCCAAATAAGCTTAC
AGCCGACCTCCCAAAAGAAATTCCAGCACCGTAAAGAGGATTGCAGGCAGATGGAAGAGGAAAGGGGGCTGGCTGGGGCTCCTGATAGACGTTAGGTATCCTCCGG
CCCATGTTCTTAAATGTACCTCGTAGTACTTACTTTGTTCAGCTTGACCATGTTTCAACATGAAGGAAAGTCTTTCGATGCCTCTTATGATATCCTGTGCCGACGTGGATAG
TTGAAGACCCTTTACTAAGCTCTGGGCTCATAGACCTGTCGACTTTTATGGATCTGCATTATTCGTGAAACAAATAATCATGGAACAATACCCTGGACCATTTATTCCCTG
CCGACCTTGAAAGTGATGCCGCAACGATCATCCAGCCACCGCCTCAGTACAACACGGCAGCCGGGCCAAGCAAAACCTGACCTTGTTAATTATTACAATGCTTTACGT
GTTTGCATTATCTGTAGCTTTTGAATAATATCAGAACTGGAGCCCTAGATTAAAAGTATCGTGTATCAACCGGGAATGGTTAGTAACTTTAGTAGTTACCTGAATATCAAC
TACCTACCTACTGCCTAGGTACTGTAGCTTGACACCTCACCTCACCATACCTGGCAGCTGACTTTCAAGACTGACCTTCATCATGTTCTTTCCATCT

> 12-B

TGCCCAGTGATAACGACTAAGTAACGGTACTTGTGATGTGGGTGGGGAGACAGTGCGGGCTCGTGGCGCATACTAGTAGATAGCTACTTATGAAACAGCGATATGCCA
GGCATGTTCTCCATTTGGTGTAGCAGTACTAATGCTGGAACGATGACGCTCTTCTTGTTGCACACTGTATATCTGTGGTCGCGAGAGCGGTGAAGCTCCTTTCTATGAGTA
TCAGTACATCAGCCCCCGCGTTACCTACATCTGAGCACGCAGCACGGAACACTACTAACTTTACGGTCATGAAGGAAAGTCTTTCGATGCCTCTTATGATATACTGTGCC
GACGTGGATAGTTGAAGACCTTTTACTAAGCTCTGGGCTCATAGACCTGTCGACTTTTATGGATCTGCATTATGCGTGAAACAAAGAATCATGGAACAATACCCTGGAC
CATTTATTCCCTGTCGACCTTGAAAGTGATGGCGCAACGATCATCCAGCCACCGCCTCAGTACAACACGGCAGCCGGGCCAAGCAAAACCTGACCTTGTTAATTATTAC
AATGCTTTATGTGTTTGCGTTATCTGTAGCTTTCGAATAATATCAAAACTGGAGCCCTAGATTAAAAGTATCGTGTATCAACCGGGAATGGTTAGTACCTGTAGTAGTTAC
CTGAATATCAACTACCTACTACCTAGGTAGGTACTGTAGCTTGACACCTCACCTCACCATACCCAGCAGCTGACTTTCAAGACTGACCTT**CATCATGTTCTTTCCATCT**

> 13-A

ATGTCTTCAAATGTAACGAGAAGCTTGAAAACGATCCAAAGAAGCAAAAGGGAAATCGTATCAAGTTTACAAAGGCGCAATATGAGTATATATCCACCCATTACATG
CTTGGCGGAGTGCTCCTTACAACCCCAGAGATATAATGATACAGGGAAGCTAATTGGAGGAGAAACTATAAACATAGTGATGTGGGAAATGCTGAAACAAACGCTAGT
TTTCCGAGCAATGTTGCGATGCCCGTGCTTCTAAGACCAAGATTGGGGCAGGGATGGCAGCTCCTAGCAGACGGTTTTCTGTTTAAGTGAGCTCCTCTCGGTGATGATTT
GCAACGAATGCCGAAACTGCCGCCTTATTATTACCTAATTCGGGGGGAGTTGCAACGAAATATCTCCGAAGCTGATGTGATTTCCCTTTATGGTATCTTGATAGAATGGTA
TTGGTGGGGAATTGTCCACTTCAACAGACCCTGGGCATGCCATTTTACATTTTGACAATCTGGATCATTATATCACAATATGTCGCAAGAGCCAATGTTAGTTGCCAGGG
TGCGAAAATTGGTTTCCGTTCAATATTTCCTTTGTTTAGTTCATCCTTCTCCGTCAGATGCCCAGATTGCAACAACTTTATCCCGAAACGATAGTGGTCGTGATAAAGTCC
TGAGCCTAGCAAAGACTTCCCAGAGTAATCCAGAGACAGAAAATAACCACGAGAGTAATACCGTTTGACGAAGCTCTTGATCCCAAGTTGGATCCCCTACTACTCTGT
CCTTCAGCACGCCCATCGCCATTCCCTTGGCCATCATGTTCAATTCGAAAATTGTAGTCATCAGTG**TAACCCGCTGGCTCACA**

> 13-B

ATGTCTTCAAATGTAACGAGAAGCTTGAAAACGATCCAAAGAAGCAAAAGGGAAATCGTATTAAGTTTACAAAGGCGCAATATGAGTATATATCCACCCATTACATGC
TTGGCGGAGTGCTCCTTACAACCCCAGAGATATAATGATACAGGGAAGCTAATTGGAGGAGAAACTATAAACATAGTGATGTGGGAAATGCTGAAACAAACGCTAGTT
TTCCGAGCAATGTTGCGATGCCCGTGCTTCTAAGACCAAGATTGGGGCAGGGATGGCAGCTCCTAGCAGACGGTTTTCTGTTTAAGTGAGCTCCTCTCGGTGATGATTTG
CAACGAATGCCGAAACTGCCGCCTTATTATTACCTAGTTCGGGGGGAGTTGCAACGAAATATCTCCGAAGCTGATTTGATTTCCCTTTATGGTATCTTAATACAGTGCATA
GACAAAAGTTCCAATATATCCCGATTATACTCTAACTACCTCGATTACACCCTACTATTTCACTCTGCAATTAACTAGGCAATATTTATTTAATTATAATAATATATATATAAA
TCTATAGAGAATTAAAAGGAGATTCTAGCTATATAGCTTAATACTTAATATAGTAGCTATTTATATAATAATACTTATTTAATTAGTTAGGTATTGATTTTATTACGTTTTTAAC
TATATTATCTTTAATTTTAGTTTATAACTTCTCTATAGCTATAATTAGCTAGCTAATTGCCTTAATAGACTTTAGATAAGTAGTAATTTTAGGATATATTTTATATATCCTTTCCT
TAAGGACTTTCTAAAGGTTTTTAATAGGGCTAAGGTTAGGACTATATAGAGGCCAGTTAATAAGAAAGATTTCTTTTCTTCTTGCCTAAAGATAGAGCTAGTTTTAAACTT
TTTAATAAGTAAATATATAGGTATTATTATACTAAAAAGTCTCTCTTTCCTTAATAAGGTTAGGAAAATAAGACTGCAGATAGGATAGAATATATTAGCCTATTACTCCCTTT
CTTTTAGATTTAAGATTCTCTAAAAGAGAGATAACTATAGATAGTTTATTATAGTTAATTATACTATAAAATATTTATAAATATCTTATAGGCTATTTATAGGCTTAGACTTTAT
TTTTATAAAGTCTTTTACCCTATAGTATTATTAAAAGAAGCTTTTAAAAAAGAAGTCTTTAAATTTATAGCTTAATAAAATAACTACTTAATATAGTTACTATCTATATAGTTA
ATTATAACCTTATTAGAAAATTACTAACTATACTAAAATAATTTATCTTTATAATAGTATTTATCTATAAAAGATTTCCTTAATTAAATAGCTTCTTTAATAAGAAATAGGCAT
TAAAGTATAAATTTATATTATATTTTCTTATTAATAAGCTAGTAATAAATTATCTTTAGTAATACCTTTAGCTATACCTAATTTATAATATCTCTATATAAAATAAAAGGGTTAT
TTTTAATAAGTATTTTAATATAACTTTTATCCTAATTTAATAGCGCTAAAGGCCTGCTAGAATATAATTTATCTTTATATAACTTTTAGGCTTAACTGCATTAAATAATCCTATA
TATTATATAATAACTTAGCCTTATACTATCTATAATATCTTTAATCTAAACTCCTAATTTATATATTATTATAAACCTTATTCTTTTATAAGAAAAATAATATACTTTTTGCCTTTA
TTAGGGCATTTTAATCTATTTAAATAATATAATGAAGTTATGGTATTAATGTTAATATTTTATTAAAAGCGGGGTTAGACCGGGAATATATCGGAACTTTCGCCGGCCCACT
GTAGAATGGTATTGGTGGGGAATTGTCCACTTCAACAGACCCTGGGCATGCCATTTTACATTCTAACAATCCGGATCATTATATCACAATATGTCGCAAGAGCTAATGTTA
GTTGCCAGGGTGCGAAAATTGGTTTCCGTTCAATATTTCCTTTGTTTAGTTCATCCTCCTCAGTCAGATGCCCAGATTGCAACAACCTTATCCCGAAACGATAGTGGTCGT
GATAAAGTCCTGAGCCTAGCAAAGACTTCCCAGAGTAATCCAGAGACAGAAAATAACCACGATAGTAATCCCGTTTGCCGAAGCTCTTGATCCCAAGTTGGATCCCCT
ACTACTCTGTCCTTCAGCACGCCCATCGCCATTCCCTTGGCCATCATGTTCAATTCGAAAATTGTAGTCATCAGTG**TAACCCGCTGGCTCACA**

> 14-A

CCTCGGTTCTGCTGTCTATTCAAGCTTGGGCCCCCGGCCCCAATTCTCACTTTCTATGCTGCCTTAACCTTCAGCGTCCAAGCCCAGAATACCAGCAGAGTGACTACGC
TATAATGGCGTACCCGGGACGATCCTCTTCCCATCCGGCCTGCTGGTTCTTGACACACTGCAATGCAGAGTATACCAGCAGTTCTGGGCTTGAGTACATTTCACTCGATA

TTCACAGGTGGCTATTGATGTTGTGGGTATAAGCCTACTGTGCATCAGCTACAATGTTTACATCTAAAACAACTAGAAGGACTAAGGAAAGGTCGTTTCACCTGTGACA
ATTCTTGATCCAAGGGTCCAGTTAGCTGAGTACTTCACTCCTGGTACTCATGGTCCGACGTCGGCTTCAGCATAGCCTTTCGTCAAGTGCTGACATCGTTGGCTGAACGA
GCTGCCTCCACTGTGCAGTCATGTTCGCGCAATTTATGTCTCAAACAAACAAAGATCTGCAGATGATGAGACGTGAATCTACTCCTCCTACAGTCACAGTTGTGACATA
CCGTCAGCTTCTGATAGTACACCCTTTGCCAAAGAACAATGCCTACGGTCTGTTGATAGTCCCTCCCACGTTTATTCATACCACACGGGATGACGAAGACTACACTCCCC
TCCAATCCAAGCGGTTTGAGCAAGTCTTTCAGCTCGGTAAACGGAATGCCCTAACTTGCTCAACGGCACATCTGGGTTTTCGGATCGAAGGACTGGGCCTGTAAGCCA
CCAAGCAAACAACTCGAGGCTTTTTGAATATGTCTCAAAGTCAAGTAATGTTCTTCAAACCTCTGTATGCTCCTTCTTTTTGCTCTTGAGGCGGTTTGATCCTATCACCTC
TAGCCAAGTTGGAAAGCTTATGTCAAGCCGCGTCTTCCTCATCATTGACCTGGGTATAAGCCATGATCATAAATGAGATGGGGCATGAGCCAGTGGGCTTCTGAATGCA
GATTAAGGCTTCTCAATCAATTGGACAAGGTTGTTCTTTCGAATACCTGTTCTGAAATAACCTTCATATCTTCATATGCGGAGGTAAATTTGCTGGTACTTGTTGACTGGCC
ATGAAACGTAGGTCCTCTGGAAGCCCTTTTTAATTACAGGTATTTTATGCAGCCTCTGTGGTCTAGTCGGTATCAATGAGTGCTGTGCACTACTTCAAGCACACACATCCA
TATCGGATCGTGCGGCCACTGGGGCCTGGGTCATCAAAAGCTGCAAAAGCAGCTGGCTAGTGCCAAGCTCACTGCGCCATTCTGCAAATCACAGCATCCAGAGAGTG
AGCAGTTGTTCTCGATTTTGCGAGAAATGCAGCCATAAAGTCACCAGGGTGTCACTTGCTGGTCTCGCTTTTAGATAGCGTCATCCACCAACATGCTGACACTGGCTCC
GGTCTTCAATCATCGTTGAGGAGGCGAGTTGTCTTGTTTCTCGTCCATAATGCTTTCACGGGAGGTATCTTCATCCCTGCTTCGTTTCTCAAAACCCATTTGTCCGTGCTCT
TCCTCGTCGAATCAAAATTACTCTGTGATGTTTTCATCACAAGACCAGTATGTGCTTAGACTCCCCTTGTGTCCCAATACAGCGATGGATTTAAATTGAGGGGCTCCAAG
AATGATATGAGCTCTAATAAAGCAGGGGTATCCGAAAGGCAGATAGGCTACTTCATCTTTTACCCCTATGTCAGAAGACTCGACAGTTCCCACCCTACGTGAAACACGA
TTGCACAAAAGCTTGGAGAGGTGTCATCCAACTTTGAGAATTCCTGGCAAAAGAAGCAGCTGTGGGTGCGAATGGCCCAGAACAATGATTGCTACACGAAGGCACTT
TCAGATTTCGAGGATCTGGGGCACGCCTTTTCTCGCGGACAGTAGGGTCTTTCTCGTTTCCATTTGTGGTGAACTTCTCTAGAAGGTAACCCCTGACGTACTACATGTTCA
CTGGACTAAAGTTCACGGTGCCAGCCAGTTGTTTGTTTTACTCTTCAGACCTCAAATCCAAACATCTGTCCTTCAAGATTGCTGATATGCTGCTGTTGGAATGAGTGCAA
CGCGGCTTCAAGCAACACACTTAACATGGTCCAGATGAGTCTAAGGGGCCCAGGCTTTGCAACACTTTTTTACGGCACGGGGCATCGGCTTCACCCAGTTCGATTTTCC
ATGCAACCGGATTGTGGAGGCTGGGGGCCTTGTGCACCTATGTTGTCATGGTGGGTTTTCACCTATAAACCATCGTTGGTTCTGGGAGGGCTCATTACATTGGGTGGCAC
ATAACCTTCTATGGACTCCTTTCTGTTAAGAGGTCAATGACAAAGTACCTTATCAAGTCACAGTTACGTTCGGTAATATAGCGGATTTCAAGCGGTCTTCCCCCTGGGGCT
TTCGGACCTCACATGGCGCAGTGAACTTTTCATCAGACACTGTTCTTGCTGGGATGTTACGCTGCCTCACCACACTTTACTCACAAACTGTTATCTCGACTTCACAGCCG
CAAGCTCGTAGATCGTCCGTAGTAGTATCCTTTTCGAGGTAAGGGTGACATCACTTCAAGCAACTCCACAATATCAAAGAAAGAAGTGATTGTGTCCAGGAACATCTTA
CTGTTGCCAGGACCTTTCTGGCCCTATCGGTTACCGCAGTTTAATCACGGGAGATTTAGTCTTAGAGAATACCAAGCGATGTCAGGACGTTATATATGGTACCAGCTCAT
GTATAGTTCAATGGGTTGTCATGATGAGGGGGTCTTCGTGGCGTTACCCCCGTGGGTATGAGAGTTTCGGGTTTCCGTTTGTGGCCAAGACCATCATGACAACTATTCGA
CAGTAACAGCGCAACCAACCTATTTGTGTATTCGACAGAAGACTTC**AGACTTCACAGCAAACA**

> 14-B

CCTCGGTTCTGCTGTCTATTCAAGCTTGGGCCCCCGGCCCCAATTCTCACTTTCTATGCTGCCTTAACCTTCAGCGTCCAAGCCCAGAATACCAGCAGAGTGACTACGC
TATAATGGCGTACCCGGGACGAGCCTCTTCCCATCCGGCCTGCTGGTTCTTGACACACTGCAATGCAGAGTATACCAGCAGTTCTGGGCTTGAGTACATTTCACTCGATA
TTCACAGGTGGCTATTGATGTTGTGGGTATAAGCCTGCTGTGCATCAGCTACAATGTTTACATCTAAAACAACTAGAAGGACTAAGGAAAGGTCGTTTCACCTGTGACA
ATTCTTGATCCAAGGGTCCAGTTGGCTGAGTACTTCACTCCTGGTTATCATGGTCCGACGTCGGCTTCAGCATAGCCTTTCGTCAAGTGCCGACATCGTTGGCTGAACGA
GCTGCCTCCACTGTGCAGTCATGTTAAGCTGCCTCACCACACTTTACTCACAAACTGTTATCTCGACTTCACAGCCGCAAGCTCGTAGATCGTCCGTAGTAGTATCCTTTT
CGAGGTAAGGGTGACATCACTTCAAGTAATTCCACAATATCAAAGAAAGAAGTGATTGTGTCCAGGAACATCTTACTGTTGCCAGGACCTTTCTGGCCCTATCGGTTAC
CCCAGTTCAATTACGGGAGATTCAGTCTTAGAGAATACCAAGCGATGTCAGGACGTTATATATGGTACCAGCTCATGCATAGTTCAATGGGTTGTCATGATGAGGGGGTC
TTTGTGGCGTTACCCCCCGTGGCTATGAGAGTTTCGGGTTTCCGTTTGTGGCCAAGACCATCATGACAACTATTCGACAGTAACAGCGCAACCAACCTATTTGTGTATTC
GACAGAAGACTTC**AGACTTCACAGCAAACA**

> 15-A

ACGGCGTTGGATATATAGATACCAATCTGATTTTATGCGCCTCTCACCTCCCCCCTTTTCTCTCAGTGTCTATGGGCGTTGGAGAAAGGGGAGAGAGAAGAGGTAGGCC
AATTTCTTTCGGCATGTACACATGATTTAATGCAGCGTTATGATTTTGCTTTTGGTTGGGTGCTTTGCCGGTGGGCGATGGGATACGCGGGAATCTAAGGAACCAGAGTTT
AAGATAAAAATTTCAAAATAGTTCGCATCGATTTCTGTGTTTAGTGAAGTGAAACGTGTTTGTTGTTCTGGTGAATGTGTTAAAATGGACTGTAATATGATCATCTCGGCA
GTCACTCTAACACGGTGCGGTGAGAGTGATTCTGGGAGCCTTTGAGGCTGGTTCACCAGCTTATCGTTCAACTTGGTCGTGTAACTAATCGGTTCATAGCAAATAGTTCG
GCATGATTACCATGAATTCTTACACTCTCTATGCCCAAGGACTCTCTATGCCCAAGGACTCTCTATACCCAAGGACTCTCTATGCCCAAGGACTCTCTATGCCCAAGGAC
TCTCTATGCCCAAGGACTCTCTATACCTAAGGACTCTCTATGCCTAAGGACTCTCTATACCTAAGGACTCTCTATGCCCAAGGACTCTCTATGCCCAACTTCTGGTCATTAT
CAGGAAGATGATATCAGATGCCGAGCCATATTTGCGATACAAGCTGCATGTAATTTACCTCGAGAGACACGAGTGTAACAAAGTAAGACATGAAAAGCGCCGAGCCA
GGGGCGCATCGAGATGTGTATTTGGGATGGTATCTCTACTGATCCAGATCTCGGCCTCCGCAGCACCAGATTGTGTTTTACCGCCCCGTATTTCCTTGTGATTTCCAACAA
GAGACAGTCTACAAAACCAGCAAAACCATGAACGATTACATTTCACCACACTGGGAGATGACTACGTCAAGGTTCGGTCTATCCGGGGCAGCATAGCCAGTTTTGGTA
GCCTCCATCTTCTTTACCACGTCCATGCCGTCGACGACCTTGCCAAAGACGACATGCTTGTTATCGAGGAAAGGTGTGGGAACAGTTGTGATGAAGA**ATTGAGAACCA
TTGGTA**

> 15-B

ACGGCGTTGGATATATAGATACCAATCTGATTTTATGCGCCTCTCACCTCCCCCCTTTTCTCTCAGTGTCTATGGGCGTTGGAGAAAGGGGGGAGAGAAGAGGTAGGCC

AATTTCTTTCGGCATGTGCACATGATTTAATGCAGCGTTATGATTTTGCTTTTGGTTGGGTGCTTTGCCGGTGGGCGATGGGATACGCGGGAATCTAAGGAACCAGAGTTT
GAGATAAAAATTCCAAAATAGTTCGCATCGATTTCTGTGTTTAGTGAAGTGAAACGTGTTTGTTGTTCTGGTGAATGTGTTAAAGTGGACTGTAATATGATCATCTCGGCA
GTCACTCTAACACGGTGCGGTGAGAGTGATTCTGGGAGCCTTTGAGGCTGGTTCACCAGCTTATCGTTCAACTTGGTCGTGTAACTAATCGGTTCATGGCAAATAGTTCG
GCATGATTACCATGAATTCTTACACTCTCTATGCCTAAGGACTCTCTATGCCCAGCTTCTGGTCATTATCAAGAAGATGATATCAGATGCCGAGCCATATTTGCGACACTA
GCTGCATGTAATTTACCTCGAGAGACACGAGTGTAACAAAGTAAGACATGAAAAGCGCCGAGCCAGGGGCGCATCGAGATGTGTATTTGGGATGGTATCTCTACTGAT
CCAGATCTCGGCCTCCGCAGCACCAGATCGTGTTTTACCGCCCCGTATCTCCTTGTGGTTTCCAACAAGAGACAGTCTACAAAACCAGCAAAACCATGAACGATTACAT
TTCACCACACTGGGAGATGACTACGTCAAGGTTCGGTCTATCCGGGGCAGCATAGCCAGTTTTGGTAGCCTCCATCTTCTTTACCACGTCCATGCCGTCGACGACCTTGC
CAAAGACGACATGCTTGTTATCGAGGAAAGGTGTGGGAACGGTTGTGATGAAGA**ATTGAGAACCATTGGTA**

> 16-A
ATGACTCTCGTGTTAAGATAGTCTGGATCGTAGAACTCCTGTGGTGGTAGACTAGCTGTCGCGACTGGAGCAGGCGTGGTGTTTCGGCTCTGTTTTGGTATGACCGAGT
TCTTCTTGCCCGTCCCCTTCTGACTCGCCCCTGGAGGCATGACCGCGGGTGTTTGAGAGTATAAATGAGCGTGGTTAGTATGCGCTTGATTGGGGGTTTAGTTGCGCGTG
GTCTATCAGATAAGATCGAAGGTTGAGTGGAAGATTTTGATGATTATTGCGACAAGGGAACAATAACAGACTGTAACGACACGGCTCTAAAGCACGCGATCCTATTAA
AGTGCGTCACGGTACAGGTTGGGCGTCGGTACGTACCTGCCCCTTGATGTTCTCAGCTGAGGGGAAACAGATGCCCCGGATGAGACCCTTGCACTTGAAGAGCAGGCA
CGGAATATGTTTAATTCCGTTCCTTTCAAGAGCAATCAGAAGCGAACAATTGAATGCCATCATCTTCAATCGTGAAGTCTCTGGTATCAGTACAAGTCACTGTCGCTAAC
GACTTTTCTCCGGATCCTTAGATTTCAATTCTGGCTGCAAAGCCCTATTCGTCGACGCTGCAACTCATTGACACTTACATAGGATGGCGACTCTCTTTGTAGTCAAACAGT
TATGAAAAACCATAATTGATACGAGAGCCGAGCTACGGCAATCCGACTTCATATACATAATCTTCAAGGAAGCCCGTTGATTTGGTTTATCCAAAGTCAGCAGCGAAAG
GCAGTATCACTAACTTCAAATTAAGGAACTTGACTTGAAATCAAGGTGTCAAAATTAACTAATCTATAGGCATAAAGTTATACGGACTTTACCCAAGTCATAGGATAAA
AGTTCTACACATGCAAATACTCCCTACCAAGACTCGGGCCTCAAAGCCACCAAGTGTTTCGGGAGGGTTAATAAACCAAGTCAACTGCATTTTTGCCAGCAATTTACTT
TGACGACCAAGATCGCGTTATTTGTCCTGGACTAGATCAATCATGCACAAAACGTGTAATGACTCGCAGTGCAACCTTTCTATAGGTGCAATGGAGTTGCACGTTCTTTG
ATAGTTCTTCTTACTCGCGGCAAACCAGAAGAAAGCCCTCAACTTCATTCAAAACTTGGTCTTTGGGAAACAAATTGACAGTGCTTTTGTCACTGTCGATAAACACCAA
CACTGGAGGCATCAGCATCACCCATTAACAAATCATTCATG**TTCAAAGAATAAATGGAA**

> 16-B
ATGACTCTCGTGTTAAGATAGTCTGGATCGTAGAACTCCTGTGGTGGTAGACTAGCTGTCGCGACTGGAGCAGGCGTGGTGTTGCGGCTCTGTTTTGGTATGACCGAGT
TCTTCTTGCCCGTCCCCTTCTGACTCGCCCCTGGAGGCATGACGGCGGGTGTTTGAGGGTATAAATGAGCGTGGTTAGTATGCGCTTGATTGGGGGTTTAGTTGCGCGTG

GTCTATCAGATAAGATCGAAGGTTGAGTGGAAGATTTTGATGATTATTGCGACAAGGGAACAATAACAGGCTGTAACGACACGGCTCTAAAGCACGCGATCCTATTAA
AGTGCGTCACGGTACAGGTTGGGCGTCGGTACGTACCTGCCCCTTGATGTTCACAGCTGAGGGGAAACAGATGCCCCGGATGAGACCCTTGCACTTGAAGAGCAGGC
ACGGAATATGTTTAATTCCGTTCCTTTCAAGAGCGATCAGAAGCGAACAATTGAATGCCATCATCTTAACTTAACTTGAAATCAAGGTGTCAAAATTCACTAATCTATAG
GCATAAAGTTATACTGACTTTACCCAAGTCTTTTTGTCATCCATAGAATAAAAGTCCTACACATACAAATACTTCCTACCAAGACTCAGGCCTCAAAGCCACCAAGTGTT
TCGGGGGTTTGATAAACCAAGTCAACTGCATTTTTGCCAGCAATTTACTTTGACGACCAAGATCGCGTTATTTGTCCTGGACTAGATCAATCATGCACAAAACGTGTAAT
GACTCGCAGTGCAACCTTTCTATAGGTGCAATGGAGTTGCACGTTCGTCGATAGTTCTTCTTACTCGCGGCAAACCAGAAGAAAGCCTTCAACTTCATTCAAAACTTGG
TCTTTGGGAAACAAATCAACAGTGCTTTTGTCACTGTCGATAAACACCAACACTGGAAGCATCAGCATCACCCATTAACAAATCATTCATG**TTCAAAGAATAAATGGA**
A

> 17-A

GTTTCGTCGCCAAAGCTGGAAGAGTCTCGACGAGAAGAACGATCCATCGCGGATACGGAATGATGAGATAATTTGCTGAATGCACGATGAAGGGCACAATCGCATTC
ACGAGGCCTGAGAGTGAGTGGTCAGTTGAGGCTTCATGTGAAGACATCCGATGAAGCAGTCAAAATTACCGAGAATCGCAAAACCGAGGTACGTTTGCATCGTAACA
CTCAACCTCGAGCTCTTCTGAGCATCCATATTGAACTCCATCACCTCAAATCCTCCATGAAAGAGTCAGATTAAAAAAAGGAAGTGAATCAAGAAAAGAGGCTTTTCA
ACAAAGAACAAATCAACCAATACTTTACGCAGCCTCAAGTCAATAAATGCAATGTAAGCTTATTTATTTAACCCTCTGCTCTGGCCGAATACGGTTTACGGAGCCCAAA
ACACCGCGCGGATAAATACGAGAGGCCATGACTCTAATAGACTACCTTCCTTACCTTAATAGGAATTATAGAGCATCTTGTTACGAATCAATTCATCACCAGCGTAAAAT
GACGTACAGAGATTGGATGGAAGAGGAAGCTGATGTAGAACCTCTTGTTGGGCCACGTGGGGCTGTTTTCAAGGGCACGATGTCGAGTGATTGGCTGTAGCGTATTGA
TTCAACGCTAAAGATCTGCGGTGAATAGTGGGTTCATGATGGATTACAGAGGCTAGAGTCATGATAGATAAAACTACATAGTACAATCTCATGAGGAGATGAAGGACA
AGATGATGATCATGGATCACAAGGGTCTCTTAGTATTTCATATCTACTCAAGATGATTGTAGCACTCCTATCAACATGATCAAGGCGATCTGCAATGACCTCGTCTTCAAT
ATCACATCGCTCGTCCTCCATGATGAGAAGAGCTCCAAATGCTGACCCTGAAGATGCATCGTTTCCATCTGCCCAGTCAGCTCGACCTGTTCTAGCTCA**GCTGAAGCCA**
TCATGGTT

> 17-B

GTTTCGTCGCCAAAGCTGGAAGAGTCTCGATGAGAAGAACGATCCATCGCGGATACGGAATGATGAGATAGTTTGCTGAGTGCACGATGAAGGGCACAATCGCATTC
ACGAGGCCTGAGAGTGAGTGGTCAGTCGAGGCTTCATGTGAAGACATCCGATGAGGTAGTAAAAATTACCGAGAATCGCAAATCCGAGGTACGTTAGCATCGTAACA
CTCAAGCTCGAGCTCTTCTGAGCATCCATGGTAAACTCCATCACCTCAAATCCTCCATGAAAGAGTCAGACTAAAAAAAGGAAGTGAATCAAGAAAAGAGGCTTTTC
AACAAAGAACAAATCAACCAATACTTTACGCAGCCTGAAGTCAATAAATGCAATGTAAGCTTATTTATTTAACCCTCTGCTCTGGCCGAATACGGTTTACGGAGCCCAA

AACACCGCGCGGATAAATACGAGAGGCCATGACTCTAAAAGAATACCTTCCTTACCTTGATAGGAATTAACGTCCCCTACCTAAAGACCGGCCACCCCTACAGACTGG
CCACCTCATTCTTAACCCAGCCACCTAATTTATATATACTTTAAACAGTTATAACTCTATTAATTTAATTCTAATTAATTTTAAACTAATACTAATTAATTTAGAATGCCTTCT
TTTACTAAAAAAGATATAAGCGTTGCACTTTAGATAGTTATAAATAGTATATCAGTAAATAAAGTAGCTAAAGCTTATAGTATTAACCGTTCTATACTTTAAGGTTAGATTA
AAGAAAGTATAATACTAAAAGAAGCCTAAAAATCTTATTAAAAGCTTTTTAATACTTAAAAAAAGAGTTTTTAAGACTAGATTATTATTTAAGTTAATCTAGAATGCCCTA
TATTATATTAGTAAATTAAAAAGTTTACCAGTAAAATTACTATCTATAATAGCTTCCTAAAAGGCATTAAAAAGAACTAGTTATAGGGCTTTTTAGGATAAAATCCTAATAT
TAAGATATTAAAAGGTAAGAAAATTAACTTTAATTAATATTATAGAGTTTCTTTTAAACTAATAAAGGCATTCTTTATACTCTTTATAATACTAGCAATACACTTAATTAAGT
AGGGGAATAGATATAATATTAATAAAGTTAAAATAATAAAAGGAATAGAAATAAACAGTTTATTCTTTAGTTATTAGTTAAAGAAGTTAGTACTTATTTATTAGTTTGGTTT
TTATACCTAGATTATAATTCTTAAGTATATTTTAGTAATTAAAAAGGTCTTAAGACCTATAATTATTTTTAAGAGAAAAATAGTTTAATAATAATATTTCCTTAAGAATTTAG
ACTTCCTAAAAGACTAAAAATTTATATATAGTAATAAAGGCTAGATAAGCAATAAATTAGCATTAATTTAATTAAAAAAAGTCTTTATTCTATTAATATAGCCTAAAAAGA
AAAATAAACCCTAACTTTTTATTCTTAATAGCTATAGAAGTTATATTATAGAAGATTTCCTCTAAAAATATTATAATAATAATATATATCTATTATTTCTTTCTATTTATATTTTT
TATATCCTTTAGCTACTAGATATTACAGTTTTTAGTTCTCTAAAGAGGGCATATTATTATCTTCTTTTAGATCTTACTTCTATCTTTAATAATAGTTATATTAATAAAATTATATT
TCTATATACTTATAATAAGGCTTAGAGAGAAGCTATTATAAAGTCTAATATAATTACTAGCTTTAAAGCTACTAGACTGTGGCCTATAAACCTAGTAAAGGTCCTAATAAA
CCTAATAGTTATAGAGATACTTTTTCCTGCTATTATAGTAAATTTACCGATAAAAAAGTAAGATTTAAGTCTCTTAAAGACTCCTTATTTATCTATATAGCTTTAGTAAGCTTT
AGGCTAAATCCTAGCTTCTATAATATAAAATCTAACTATTAGGCTGCTTTTTAAGAAGATTAGCAGTTAATTAAACTATTATAACTTTAATATTAAGTAATAAAATAGAGAA
ATAAGTATTTTATAGTAAAAAAATAAAGAAAATTAATTAAAATAATATAAAAAAGTTATTTATAACTTAAATATAGAGTTTATAAAGATTCTAGTAATAAAGAAAGCTTAT
AAATAGATATAAAAAACTTTATAACTAAAAAAAAATAATAAATAAAATTAAGAAATTAAAACTTAAGGATCTTTATATTAATTTCTATATAAATATTTACTAACTGAAGTTTT
ATTTAATATAAAAAATATAGTGTTATAATAATTTATTAATTAATTATAATATTAGGTTTTTGGTGTTTTGGCTAAGGTGGCTAGTCTGTAGGGGTGGCCGGTCTTTAGGTGGG
GGACGTTATAGAGCATCTTGTTACGAATCAATTCATCACTAGCGTAAAATGACGTACAGAGATTGGATGGAAGAGGAAGCTGATGTAGAACCTCTTGTTGGGCCACGTG
GGGCTGTTTTCAAGGGCACGATGCCAAGTGATTGGCTGTAGCGTATTGTTTCAACGCTAAAGATCAGCGGTGAATAGTGGGTTCATGATGGATTACAGAGGCTAGAGTC
ATGATAGATAAAACTACAGTCTCATGAGGAGATGAAGGACAAGATGATGATCATGGATCACAAGGGTCTCTTGGTATTTCATATCTACTCAAGATGATTGTAGCACTCCC
ATCAACATGATCAAGGCGATCTGCAATGACCTCGTCTTCAATATCACATCGCTCGTCCTCCATGATGAGAAGAGCTCCAAATGCTCACCCTGAAGATGCATCGTTTCCAT
CTGCCCAGTCAGCTCGACCTGTTCTAGCTCA**GCTGAAGCCATCATGGTT**

> 18-A

GCGTGTTGACCCATTTCTGCCAACACCCTCCTCAAATCAGTACTGTCTGACTTGGTAGCACAATGTGACATCTAAAAGCACTAGTTATGTGTAATTACATGCACACTGT
ATCACAATATCACATATCGGTCGTGGGATTAGGGTGCGAGGGGCGTTGACTAGATTGAAGCTGAACGATGTTTTGAGAAACAGTGGCATTAATATGGCTGAATGTGACC

AACTACAATGCCCTATGGTAATTTCAGTTCTCCACCTGGGTCGACCTTATTATTTTAATATTAGTTATATAATATTATATAATAAACCGGCACTATATAAAAAGGAGAAAAA
GACTTAATGTATTTACTGAAGAATATAGTTAAAAGATAGCATAAAAATTAAATTTTAAAATCAAAAGGCTTCAGAAATGCAAGAAAAAGTGATTTTTATAAAACTTACTG
TAGGGCCTGGTAAGCCGCGATCCTCCCTCGTGATTGTGGCCTAATTTACACATATCGACTTTGACAAGGGTGCTTCCAGGTTTATGAAAGTATTAGTTCTTTTGTACCCAA
TTTTTCCAAGTAAACGTGAGCCCCCTTACCTCAATCGTTTGAAACATGCCAGGGTTTTGCTTTTTTCCACCTGAAGGCGTCATGGCCGAGTTTGATCAAAACATTACAGA
CCAGACCTACTGTAACTATAAAACATAGCACATTCCTGTTATCCAAGAATGGAGTTGGGAATCATAAATATCTGGTCCGATCTATTTCCTGTATATCAATAATCGCCTTCTC
AAACATGTCTATCACTATCGACAGAAATCTCTCTAACAGCATGCCCAACGAGAATATTTCCGCCGAGAGCATTCGACTCCCTCTCGACGTAGTCGAGTACTTATGCTCAC
AATCTAAGAACAGTACGGGACACACCCAGAGGCAGAGACTTGGAAACCCTGCGCCAGTTGGTCAGTAATTTGTCCCAAAATATCCCACCAAATTTACAGGCATAGGTC
TCGGGGGCTTTCTCCTTTGTGCAACACCCGTCTCACTGTCACTGCTCGAGCTCCAAGGAGTAGGAGGTTTTGCTGCTGCTGCAAATATGTAAGGAAAGCCGCTGATCAC
CGGCCAAACTTGCTAACCGTCGACAGTGGTGCCTATCTGGGACTCGGCAGTGTGATGCTTATCCTTGGAGGGATTGGCGAGTGGGTTCTGGGTAAGTTAGCTGAACTCA
TATGATCTACCGACACTCACATATCAGGAAACACGTTTTCTTCTGTAGTATTCTGCCTTTTTGGCGGCTACTGGTTGACTTTAGGAATAACGATCATTCCTGGATCTGGTGC
ATATGGCATTTACTCAACTACCGAATCAGCTGTAGATGTGCTTGATAATCCTCAGTTCTACGCTACGTTTTCTTTCATCCTCGTTGCTATGACGATACTCTGCGCCCTTCTTA
CAGTTGCAAGTATTCGAACCAATATCGTTTGGTTCTTTATGTTCGTTCTCCTCACTACTTACTGTAAGATTTCTTATGCCTTGTCTCCTCATTGTAACGACTGACAGGGGTCT
TTTTATAGTTGGCTGTCTTGCTGGTTCGTTTTTTACATTCATGGAAAAACCTTCAAGTGCTCTTTTATTACAAAAGGTGGGTGCTGGGTTTCTTTTTGCAGCGACGATACTG
GGTTGGTACATGTGGTTTGCTCTTATACTCTTATCCGTCAACTTCCCGATTTCTCTACCTGTGGGTGAGCTTTCGTCAATCGTTCCCGGCCGGAACAACCAGGCAGAAATG
AAAAACACGAAAGATGCTTAAGCTCTTTACTCGGGCAGTTATTCGGCTGTATAAAACCAGAGTTGAAAGTAGGCGGGTACGATTGTTTAGCTCGATGGTTTCAAAAGCT
CAGTAGTGGATAAGTTCCAGTGCATCGTGTAGCTGGAACAATTTCTATTGTATCTTTGTAAATATTCTGGTTATTTAGACGAAAGCAAGAGATTTAATGTAATCTTCAATAT
CCCTCCAGT**GGCTAAATCAAGTTGCG**

> 18-B

GCGTGTTGACCCATTTCTGCCAACACCCTCCTCAAATCAGTGCTGTCTGACTTCGTAGCACAATGTGACATCTAAAAGCACTAGTAATGTGTAATTACATGCACACTGT
ATCACAATATCACATATCGGTCGTGGGATTAGGGTGCGAGGGGCGTTGACTAGATTGAAGCTGAACGATGTTTTGAGAAACAGTGGCATTAATATGGCTGAATGTGACC
AACTACAATGCCCTATGGTAATTTCAGTTCTCCACCTGGGTCGACCTTATTATTTTAATATTAGTTATATAATATTATATAATAAACCGGCACTATATAAAAAGGAGAAAAA
GACTTAATGTATTTACTGAAGAATATAGTTAAAAGATAGCACAAAAATTAAATTTTAAAATCAAAAGGCTTCAGAAATGCAAGAAAAAGTGATTTTAATAAAACTTACT
GTAGGGCCTGGTAAGCCTCGATCCTCGACTAACAGGGGTCTTTTTATAGTTGGCTGTCTTGCTGGTTCGTTTTTTACATTCATGGAAAAACCTTCAAGTGCTCTTTTATTAC
AAAAGGTGGGTGCTGGGTTTCTTTTTGCAGCGACGATACTGGGTTGGTACATGTGGTTTGCTCTTATACTCTTATCCGTCAACTTCCCGAGTTCTCTACCTGTGGGTGAGC
TTTCGTCAGTCGTTCCCGGCCGGAACAACCAGGCAGAAATGAAAAACACGAAAGATGCTTAAGCTCTTTACTCGGGCAGTTATTCGGCTGTATAAATCCAGAGTTGAA

AGTAGGCGGGTACGATTGTTTAGCTCGATGGTTTCAAAAGCTCAGTAGTGGATAAGTTCCAGTGCATCGTGTAGCTGGAACAATTTCTATTGTATCTTTGTAAATATTCTG
GTTATTTAGACGAAAGCAAGAGATGTAATGTAACCTTCAATATCCCTCCAGT**GGCTAAATCAAGTTGCG**

> 19-A

AGTATCTGAACCAATGCCACTTGTGGAGTGTTATAGACAATGGGCTTGGACAAGTTCAAAACATAGGTTCAACAGACCATTCTCAGGTACAAGGCCTTTTCCTTCCTGG
GCGTCTTTGGTGTCTTCAAGCCCATCCTGAGTTTGATCCAGCAGTTATGGGACAGGTGCTAGACCTGACTAGATCTGAATTGACAGATGAGGAATACCGAAAGGCAAA
GGCTCATAACCAACAAAAGCTTGACCAACAAGTCGCACTAGAATCATTAGTTAATTTATTCTAGAATAAGATATTTATAATATTCCCTTTATATTATAGAATATATAGTTAT
AACTATATAATATAGTAAAATATATCTTAAGAAAATAGTTTTAATTTATCATTATATTTTACAAGTAAAGTTAAATAATAACATACTTATACTTCTTTTCTTTTTAAGTAAAAT
ATAATTATAAATAATAGGAAGATATAAAATTATCTATAATATTTTATAATATATTTCTCTCCTTTTAATATACTAGAATAAAATATATTTTACTTTATTAGGCCGCTTTACAGCG
CTTCATATTTAAAGATAACAAATTGCAAATATATAAATACTTCTTTCCTTTCCCCTCCACCAGGTCAGGGGTATTCTTTCAAGTATTACAAACATCTTGATTCTTAGAGGTA
CTTTACCGCCCCTGGTAATATTTGTTAAGCCCAAAAGCAAGTAAAGGTTACGATTCAGTCTATGCACATTATGCCCACACTGCCACATGCCACGTCATCACTTACGATGA
GAAGCTTACAATAGCTAGAAATGCCTTGAAGCCTTCAACGCTGAACGGGCTTCCTTTCCCAACTCTCAAGCCAGATATGGTATTGGAAGTAATATCCAACAGAACCCCC
TCACCTGACTTTAAAATAACCGGAATATAGTGGATGCCAATAGCATTGAGGAAGGAAACGCAATCATCGATGCCTATATGGGACAGGATCGAATCATGAAAGAAGTAA
CTAGGGATCAGAAATAAGAGGCTTACTGGGAAATACTCGCTGAGTTCCCAGTAAGTATGGCAAAGAACGATTTTATAAGTCCTTCTGGGTAAGCGGCTTTCGGCATAAT
TGAAGAACATGTATTTCTCAACAACTTTACAAGCGCTTAAGATTGACTTATTTAATTTAAGGCTGAATTACGCTATATAGTGCCGATATAGAGCCCGCTTTAGCTTCTCAA
ATAGGATATGTTATTATGACGACTTTTGCAAAAATAAATATATTTTAAGAGTGCTTTGTTAATTTCTTGCCTAGGGAGTTTTGACATAGCGTGTCTGTAAGAATGACCAGCA
TCTAAACACAGCGTAAAACAGAATCCGATCAGTGGGCTTCGTAACAGTTTTGAAACTCAG**CACGACTAGTTCCTTCT**

> 19-B

AGTATCTGAACCAATGCCACTTGTGGAGTGTTATAGACAATGGGCTTGGACAAGTTCAAAACATAGGTTCAACAGACCATTCTCAGGTACAAGGCCTTTTCCTTCCTGG
GCGTCTTTGGTGTCTTCAAGCCCATCCTGAGTTTGATCCAGCAGTTATGGGACAGGTGCTAGACCTGACTAGATCTGAATTGACAGATGAGGAATACCGAAAGGCAAG
GGCTCATAACCAACAAAAGCTTGACCAACAAGTCGCACTAGAATCATTAGTTAATTTATTCTAGAATAAGATGTTTATAATATTCCCTTTATATCATAGAATATATAGTTA
TAACTATATAATATAGTAAAATATATCATAAGAAAATAGTTTTAATTTATTATTATATTTTTTAAGTAAAGTTAAATAATAACATACTTATACTTCTTTTCTTTTTGAGTAAAAT
ATAATTATAAATAATAGGAAGATATAAAGTTATCTATAATATTTTATAATATATTTCCGGAATATAGTGGATGCCAATAGCATTGAGGAAGGAAACGCAATCATCGATGCCT
ATATGGGACAGGATCGAATCATGAAAGAAGTAACTAGGGATCAGAAATAAGATGCTTACTGAGAAATACTCGCTGAGTTCACAGTAAGTATGGCAAAGAACGATTTTA
TAAGTCCTTCTGGGTAAGCGGTTTTCGGCATAATTGAAGAATATGTATTTCTCAACAACTTTACAAGCGCTTAAGATTGACTGATTTAATTTAAGGCTGAAATACGCTATA

TAGTGCCTATATAGAGCCCGCTTTAGCTTCTCAAATAGGATATGTTATTATGACGACTTTTGCAAAAATAAATATATTTTAAGAGTGCTTTGTTAATTTCTTGCCTAGGGAGT
TTTGACATAGCGTGTCTGTAAGAATGACCAGCATCTAAACACAGCGTAAAACAGAATCCGATCAGTGGGCTTCGTAACAGTTTTGAAACTCAG**CACGACTAGTTCCTT
CT**

> 20-A

AAAGCAGTGGCGAACATCAACTTGACTACTTTCAAGTCGTCGGGACCGATGATGGTGGTATCTTGCAGAGAAATTCGCCCGCATCTATGTCGAGACAGGATTGCAATG
GAGCCATATAACTTATCGACGTTGCTTCGAATCATTTCCATATCAAGGAAAGGATGTGAGATATTAATCTCTTCTTCGTAAACATTGCAAAGCCGCAGCGCCTCTGGTCTG
TCGATCAGCCAAATTGGATCGACAGGGGGCTCATTCGATAGCGCAGTTTGTGGTGTAGGCGATGGCCGTCGGGTATTATTTCCAGCAGTCCATGCATCGCTATTATCGTT
GTGCGGTTGAGAGATACCTCGAGCAAGAAGGCTTGATTGTGCAAGGCTAATGTTGAACTTGTAGTGAGCAGTTGCAGGTGTATAGATGCTTGCTGCGCCGGCTTCGGAG
CTGCGGTTACTGTCGACTGAGTAGGTATTTAGAATAGGGGGGGAGTTGAGGGATACGTTTAGATCAGTATTCATACTTTGTTGCATAGGCACATTGTCCTGACCTGGAAC
CTGTCGAGGAGCTGAAGAATTTGTAAGAGGTTGCGGCGGTGACGGCGAAGGCGACGAAGCAAGATGCGTAAGGTCTGAGTTGTTGAGAATGGCTTCTTCGATGCTTGT
GTCAAAGGCAACTCTAGTAGATGAAGGATTTGTTGGGGCAGGCGGCTGGGCTGGTGATGACCGGATGTGATCTTCAATATCAGCCAACCTCTTGAGAATCACCCTTGTC
TCATTTGTCTCACTATGGGGGTCAGTGACATTTTGTCTAAAGCTATACCCAAGAAGAAACAAGGCTTACAGAGTTGTCCCTCGGCGACGGGGCACATGTGGAGGAAAC
GTACCTGTTTGGTGTAAGTTGTTTTCAGAGCGCGACCATGGCGAGAATAACTTACAATCCTTACCGGCTTTAGCGCATTGGCTACATGGACGTTCGCTGTTACATTTAGTT
TTGCGCTTCTTGCATTCGTAACTACATGGGGCACAAGAATGAGCTTCTAGACATCCAAGTGTCCAAGTAGCGAAGAGGTACCAGGCGAGATTTGTATATTGGCC**TCGTT
TTCGTTTTGCTG**

> 20-B

AAAGCAGTGGCGAACATCAACTTGACTACTTTCAAGTCGTCGGGACCGATGATGGTGGTATCTTGCAGAGAAATTCGCCCGCATCTATGTCGAGACAGGATTGCAATG
GAGCCATATAACTTATCGACGTTGCTGCGAATCATTTCCATATCAAGGAAAGGATGTGAGATATTAATCTCTTCTTCGTAAACATTGCAAAGCCGCAGCGCCTCTGGTCT
GTCGATCAGCCAAATTGGATCGACAGGGGGCTCATTCGATAGCGCAGTTTGTGGTGTAGGCGATGGCCGTCAGACATCCAAGTGTCCAAGTAGCGAAGAGGTACCAGG
CGAGATTTGTATATTGGCC**TCGTTTTCGTTTTGCTG**
